# Understanding Sources of Variation to Improve the Reproducibility of Radiomics

**DOI:** 10.3389/fonc.2021.633176

**Published:** 2021-03-29

**Authors:** Binsheng Zhao

**Affiliations:** Department of Radiology, Columbia University Irving Medical Center, New York, NY, United States

**Keywords:** radiomics, lung cancer, reproducibility, variability, CT acquisition, tumor segmentation, feature extraction, quality control

## Abstract

Radiomics is the method of choice for investigating the association between cancer imaging phenotype, cancer genotype and clinical outcome prediction in the era of precision medicine. The fast dispersal of this new methodology has benefited from the existing advances of the core technologies involved in radiomics workflow: image acquisition, tumor segmentation, feature extraction and machine learning. However, despite the rapidly increasing body of publications, there is no real clinical use of a developed radiomics signature so far. Reasons are multifaceted. One of the major challenges is the lack of reproducibility and generalizability of the reported radiomics signatures (features and models). Sources of variation exist in each step of the workflow; some are controllable or can be controlled to certain degrees, while others are uncontrollable or even unknown. Insufficient transparency in reporting radiomics studies further prevents translation of the developed radiomics signatures from the bench to the bedside. This review article first addresses sources of variation, which is illustrated using demonstrative examples. Then, it reviews a number of published studies and progresses made to date in the investigation and improvement of feature reproducibility and model performance. Lastly, it discusses potential strategies and practical considerations to reduce feature variability and improve the quality of radiomics study. This review focuses on CT image acquisition, tumor segmentation, quantitative feature extraction, and the disease of lung cancer.

## Introduction

Radiomics refers to the determination of tumor imaging phenotypes by extracting and analyzing a large number of quantitative image features, a.k.a. radiomics features (1–3). Unlike molecular- and tissue-based analyses, radiomics strives to characterize differences in tumor phenotypes based on non-invasive radiographic images that can be routinely obtained from clinical practice and clinical trials. Radiomics can capture the heterogeneity of a whole tumor and tumor metastases in multiple body sites and their surrounding tissues, and it can be used to monitor changes in tumor biology (e.g., mutation status) over time. Thus, radiomics is promising to be capable of addressing key issues across the continuum of cancer care.

The hypothesis underpinning radiomics is that disease processes, which produce histopathological and genetic alterations, also manifest in characteristic phenotypes that can be captured by radiographic images. Qualitative visual interpretation of CT features has been used by radiologists in making routine diagnoses for decades, such as lung nodules with spiculated edges indicating malignancy and an enlarged tumor size (diameter) post-therapy indicating a worse prognosis for the treatment. However, the big moment for cancer imaging phenotype was the 2007 article on the reconstruction of global gene expression profiles of hepatocellular carcinoma (HCC) using predefined imaging traits assessed qualitatively by radiologists on contrast-enhanced CT (CECT) (4). A new radiogenomic venous invasion scoring system, derived from three imaging traits (internal arteries, hypodense halos, and tumor-liver difference) on CECT in HCC, was reported to serve as a noninvasive imaging biomarker for histological microvascular invasion, a tissue biomarker associated with early disease recurrence and poor overall survival (5). While human eyes have an incredible ability to recognize both local and global patterns, visual interpretations can be subjective and prone to variation especially when evaluating subtle differences. Radiomics can objectively discern clinically relevant information that human eyes cannot even perceive. Indeed, a fast-growing literature shows the great promise of radiomics signatures (radiomics features and models) as a “virtual biopsy” to assist in cancer diagnosis and prognosis, treatment plan, patient stratification, and assessment of tumor response to therapy. The current status of CT-based radiomics in lung cancer has been well summarized in a recent collection of review articles [e.g., (6–17)].

**Figure 1:**
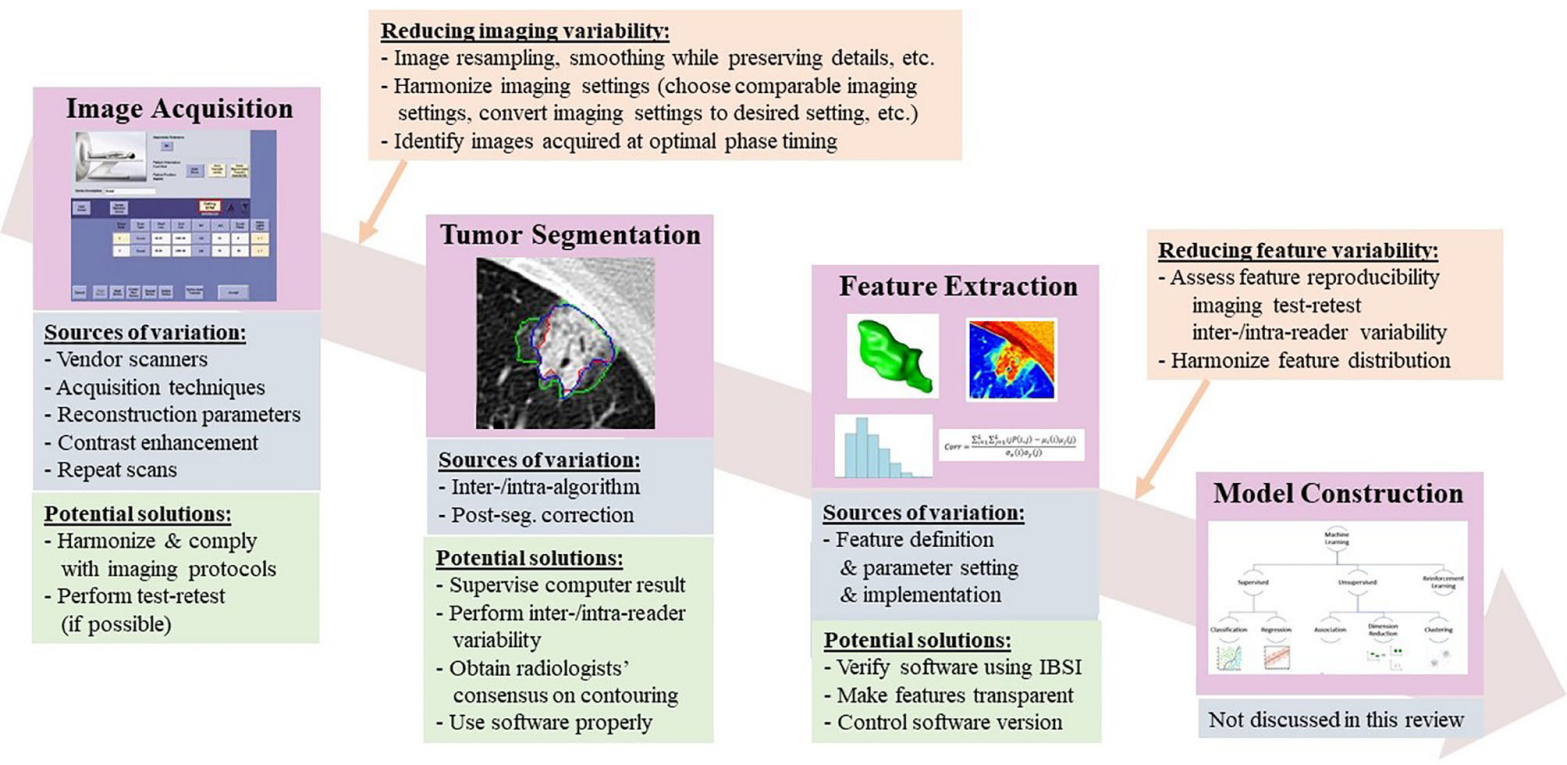
Radiomics workflow, along with sources of variation and potential strategies to reduce feature variability.

Radiomics features are well defined, and some are even intuitive (in line with expert radiologists’ visual interpretation). Radiomics analysis is a favorable approach for studying tumor imaging phenotypes because performing it requires a relatively small number of patients to train models compared to convolutional neural networks (CNNs), and sometimes it yields explainable analysis results as well. However, multiple sources of variation in every step of the radiomics workflow create an intrinsic methodological weakness that has been recognized since the earliest days of radiomics analysis (18, 19) (**Figure 1**). For instance, radiomics features can be sensitive to heterogeneous image acquisition settings (scanners, scanning techniques, and reconstruction parameters). Unknown ground truth of tumor boundaries can introduce uncertainty into features derived from segmented tumors. Despite an explosive increase in the radiomics literature, this research frequently fails to adequately consider sources of variation and reports isolated results not validated by replication in external data sets (20). The resulting concerns about rigor and reproducibility slow the pace of innovation in radiomics and limit its translational potential.

Recognizing the need to evaluate the scientific merit and clinical utility of radiomics studies, a group of scientists proposed a radiomics quality score (RQS) in 2017 (21) which evaluates a set of essential components in the radiomics workflow, starting with the quality of image protocol and ending with the availability of open science and data. A maximum of 36 possible points is awarded by scoring each component’s accordance to the suggested guidelines, with more important aspects earning more points. While the RQS is not perfect, it does establish a set of practices that can facilitate clinical translation of radiomics research. It also highlights the weakness of the current literature: the mean RQS scores of published radiomics studies are low (<10 points) (22, 23), indicating inadequate scientific quality.

**Figure 2:**
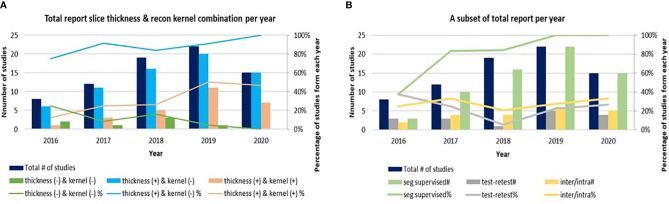
**(A)** A trend of radiomics studies reporting image acquisition parameters of slice thickness, reconstruction kernels and both. **(B)** A trend of radiomics studies reporting re-imaging, re-segmentation and supervised segmentation. Due to the small number of radiomics studies published in 2014 and 2015, those studies are excluded from the graphs.

The quality of radiomics studies has recently improved thanks to community-wide efforts to explore and reduce variability in medical imaging and to promote the translation of quantitative imaging biomarkers into clinical practice and clinical trials (24–27) (**Figure 2**). **Figures 2A, B** are drawn based on a research team’s recent literature search for the CT-based radiomics studies in lung cancer (6), which we supplemented with studies published as of July, 2020 as well as information about imaging parameters (slice thickness, reconstruction kernel) and segmentation (inter-/intra-variability, software, result supervised or not) (**Table 1** in **Supplementary Material**). Although previous imaging studies have shown the effects of slice thickness and reconstruction kernel on computed features, between ~5% and ~25% of radiomics studies prior to 2020 did not report their study imaging protocols (**Figure 2A**, green color). Most of those who reported their imaging protocols only included the slice thickness information (**Figure 2A**, blue color). It is good to see that the trend of reporting both slice thickness and reconstruction kernel increased from 10% in 2016 to 50% in 2020 (**Figure 2A**, pinkish-orange color). Nevertheless, half of the radiomics studies still do not seem to have considered the effects of reconstruction kernel on radiomics features, especially texture features. The percentages of studies that performed imaging test-retest and inter-/intra-segmentation have been stable over the years, varying between ~20% - 40% (**Figure 2B**, gray and yellow colors). All radiomics studies published in 2019 and 2020 reported human supervision of tumor segmentation (**Figure 2B**, green color), an important step to ensure the accuracy of segmentation, while only ~40% studies did so in 2016.

A valid quantitative imaging biomarker must be informative, or sensitive to underlying biology, as well as reproducible and reliable across various image acquisition settings and quantitative methods. It is essential to understand and regulate the sources of variation to ensure that consistent high quality images can be meaningfully analyzed and biological information can be reliably extracted by advanced quantitative methods. This article starts with image acquisition, then considers tumor segmentation and feature extraction. Readers who are interested in machine learning for radiomics are referred to (10, 16, 17). From the point of view of image analysis, in each of the following sections, it first illustrates how radiomics features can be affected by various factors using demonstrative examples, then reviews a number of published studies exploring sources of variation and offering increased reproducibility of radiomics features and models. Lastly, it discusses potential strategies and practical considerations to reduce feature variability and improve the quality of radiomics studies.

## Image Acquisition

Radiomics signatures aim to characterize the phenotypes of tumors and surrounding tissue using radiographic images. They can be sensitive to image quality governed by image acquisition settings, or the constellation of factors involved in acquiring the images, which include (but is not limited to) scanner equipment, acquisition techniques, reconstruction parameters, and contrast administration.

Radiomics studies have mostly used retrospective analysis of imaging data from historical studies and clinical trials that were not designed for quantitative feature analysis of tumors. Many of the images studied were acquired in clinical trials to make simple measurements of tumor diameter on CT images, which did not demand a high degree of standardization in image acquisition parameters. Because these datasets are now being radiomically analyzed retrospectively, and new data sets are being acquired prospectively, the importance of the degree of variation in CT acquisition needs to be determined.

Pioneering efforts revealed that imaging variables, such as repeat CT scans (28), imaging reconstruction slice thicknesses and kernels (29), and scanners (30), could affect the computed values of radiomics features. These studies inspired intensive investigations in feature variability and reproducibility, which have confirmed the initial findings and extended them to broader research areas. Investigations on the sources of variation in CT image acquisition have mainly focused on one or combinations of the following factors: test-retest (28, 31, 32), vendors’ scanner (30, 33–36), tube voltage and current (37–41), pitch (36), field of view/pixel spacing (42–44), reconstruction kernel and slice thickness (we do not here distinguish between slice thickness and slice interval, the real physical distance between any two adjacent images) (29, 31, 38, 39, 45–47), contrast administration (48–50), and 4D phases (51, 52). In the following subsections, a number of studies exploring sources of variation in image acquisition is reviewed, followed by a discussion on potential strategies and practical considerations to reduce variability in image acquisition.

### *In Vivo* “Same-Day” Repeat CT Studies

Radiomics features derived from tumor images from two CT scans performed on the same day or during a short time period can be different due to factors such as the patient’s relocation, breath holding, and organ movement, even though no biological changes would be expected to be discernable during such a short time period. The reproducibility of radiomics features on repeat CT scans must be demonstrated in order to establish the reliability of radiomics models built using these features.

#### Repeat CT in Lung Cancer

Early radiomics studies already took into account the effects of repeat CT imaging and re-segmentation on features’ reproducibility (3, 28, 53), thanks to the availability of The Reference Image Database to Evaluate Therapy Response’s Lung CT Collection (RIDER Lung) (54, 55). RIDER Lung is a unique, publicly available same-day repeat CT image dataset that allows exploration of the reproducibility of quantitative methods, including segmentation and feature extraction, for lung cancer studies. This dataset consists of 31 non-small cell lung cancer (NSCLC) patients’ repeat CT scan images reconstructed using 1.25 mm slice thickness and the lung kernel. Unfortunately, RIDER Lung is suboptimal as test-retest for radiomics studies because CT images in the majority of clinical studies were not reconstructed using 1.25 mm slice thickness and the lung kernel.

**Figure 3:**
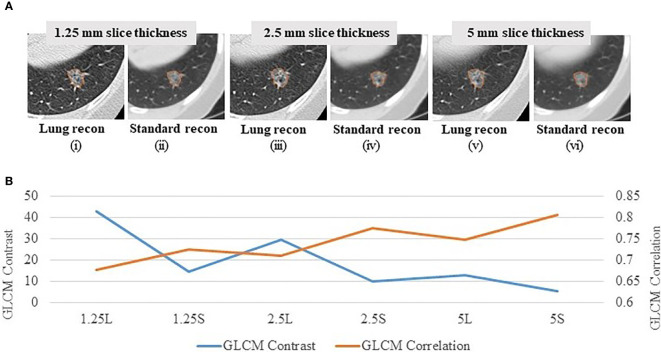
Effects of imaging parameters on radiomics features **(A)** A lung tumor captured on one CT scan reconstructed at 6 different imaging settings: 1.25 mm slice thickness with the lung reconstruction algorithm (sharp image) (1.25L) (i) and the standard reconstruction algorithm (smooth image) (1.25S) (ii); 2.5 mm slice thickness with lung reconstruction (2.5L) (iii) and standard reconstruction (2.5S) (iv); 5 mm slice thickness with lung reconstruction (5L) (v) and standard reconstruction (5S) (vi) (31). **(B)** GLCM Contrast (blue color) and Correlation (orange color) features computed at the 6 corresponding imaging reconstruction settings. The bigger the value of Contrast, the more heterogeneous the tumor. The greater the value of Correlation, the more homogeneous the tumor.

**Figure 4:**
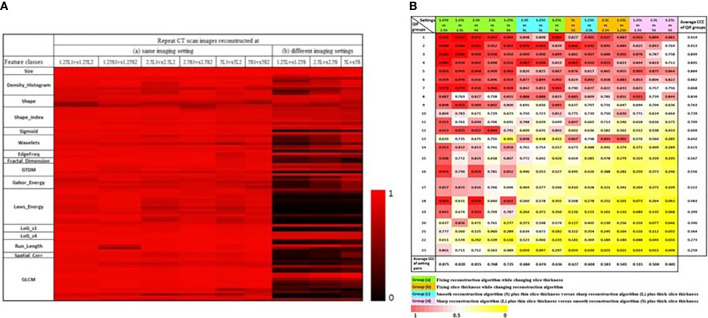
**(A)** CCC heat map of radiomics features. The CCC (0 to 1) of the studied 89 radiomics features were computed from same-day repeat CT images reconstructed at (a) six identical imaging settings or (b) three different imaging settings. The brighter the red color, the higher the CCC values (i.e., the more reproducible) of a feature computed for the repeat scans (31). **(B)** CCC heat map of 23 non-redundant radiomics feature groups (rows) under 15 inter-setting comparisons (columns). Columns are arranged in descending order according to the average CCC of the inter-setting comparisons. Rows are arranged in descending order according to average CCCs of non-redundant feature groups (45).

In order to explore reproducibility and variability in radiomics features due to re-imaging at multiple acquisition settings with same or different imaging parameters, investigators published a pilot study on 89 commonly used radiomics features using same-day repeat CT scan images reconstructed at six imaging settings/series: a combination of three slice thicknesses (1.25 mm, 2.5 mm, 5 mm) and two reconstruction kernels (lung (L): a sharp kernel; standard (S): a smooth kernel) (31). These settings cover the CT acquisition parameters widely used in lung cancer oncology trials and clinical practice. **Figure 3A** shows an example of a lung cancer tumor captured on a CT scan that was reconstructed using six different imaging settings. Given the same slice thickness, tumor heterogeneity can be better seen on sharper images than on smoother ones. The curves beneath the tumor images show the values of two popular GLCM features, Contrast (**Figure 3B**, blue color) and Correlation (**Figure 3B**, orange color), calculated under each imaging setting. The bigger the value of Contrast, the more heterogeneous the tumor. The greater the value of Correlation, the more homogeneous the tumor. In this example, the value differences were caused by different imaging reconstruction parameters, not by the tumor’s underlying biological effects. The study found that the radiomics features were generally reproducible when calculated between two repeat scans reconstructed using the same imaging setting. This is indicated by quite uniformly bright red areas (high concordance correlation coefficient (CCC) values) in **Figure 4A(a)**. However, a substantial amount of variability was observed within the same slice thickness when using standard or lung reconstruction kernels, generating smooth and sharp images respectively, as indicated by large dark areas (low CCC values) mostly centered at the texture features [**Figure 4A(b)**]. The authors’ conclusion that smooth and sharp reconstructions should not be treated as interchangeable for radiomics studies has been confirmed by other independent studies (29, 36, 56).

#### Repeat CT in Rectal Cancer

RIDER Lung was a very well controlled clinical study in which the two repeat non-contrast chest CT scans were performed within 15 minutes using the same imaging protocol on the same scanner. Other radiomics studies also reported good reproducibility when testing their quantitative features using RIDER Lung [e.g., (32, 36)]. However, it is possible that repeat CT scan images of other organs may cause different magnitudes of feature reproducibility. A study found much lower feature reproducibility in rectal cancer than in lung cancer (32). The investigators collected repeat CT scan images from 40 rectal cancer patients in a clinical setting; the interval times between two repeat scans ranged from 5 to 19 days. They reported that only 9/542 features had CCC >0.85 in rectal cancer, whereas 446/542 features had higher CCC values for the test-retest analysis of the RIDER Lung dataset. However, this is not surprising because the longer interval times between the two repeat scans in the rectal cancer study, the possible use of different imaging settings for two repeat scans, and presence of more noise in rectal images could all contribute to the decreased reproducibility.

#### Four-Dimensional CT (4D CT)

The same-day repeat CT images in the RIDER Lung collection were acquired with each patient holding their breath. Radiotherapy scan images, however, are often acquired under free breathing of the patients. Respiratory motion can cause changes in tumor location, volume, shape and intensity (57) leading to more uncertainty of target tumors and enlarged margins in the delineation of the treatment volumes. To decrease the amount of radiation exposures to healthy tissues, an emerging modality of gated or 4D CT imaging has been developed and used in radiation treatment planning (58). During a thoracic 4D CT study, multiple CT images are acquired over a period of at least one full respiratory cycle (8 or 10 phases) at each table position. Moving the table and synchronizing the scans according to the patient’s air-flow-volume curve, a spatial-temporal 4D CT dataset can be acquired. After being sorted, the motion-reduced 3D CT image series acquired at each respiratory cycle can be generated.

A recent study investigated respiration-related 4D stability of radiomics features across 8 individual respiratory phases for NSCLC (59). Eight hundred forty-one features were extracted from all individual phases of each patient. The relationship between individual coefficients of variation (COVs) and tumor motion magnitude was also inspected. The study found that some features (e.g., skewness, many GLDM features) were sensitive to respiration, whereas others (e.g., shape related features, many GLCM features) were not. The study did not observe a clear trend between the feature stability and the motion magnitude due to respiration. In the second part of the study, the value of utilizing 4D stability to preselect radiomics features to build prognostic prediction models for overall survival in early-stage NSCLC radiotherapy patients was explored. By comparing the performance of the models built with and without 4D stability feature preselection, the study showed an improved prediction performance with the preselection. Other studies in radiation oncology also suggested using phase images of already acquired 4D CT data as an alternative way to determine and remove unstable radiomics features prior to radiomics model construction when test-retest images were not available (51, 52, 60).

### Radiomics Phantoms

Due to concerns such as radiation dose to patients, comprehensive investigations of image acquisition’s effects on radiomics features have to rely on phantom studies. However, there is a significant disparity between tumor phenotypes that are seen in patient clinical CT images and traditional physical phantoms (e.g., simple shape, homogeneous density) (61).

#### Credence Cartridge Radiomics (CCR) Phantom

A group of medical physicists designed the CCR phantom to assist in exploring intra- and inter-scanner robustness and reproducibility of radiomics features (30). The CCR phantom embraces ten cartridges of an equal size of 10.1×10.1×3.2 cm^3^, each filled with different materials in different patterns. The phantoms were scanned on 17 scanners from the four major CT vendors at multiple medical centers using their local thoracic imaging protocols. Both histogram-based and texture features were extracted using the open source radiomics software package of IBEX (62). The study results showed that the phantom’s dynamic density range covered that observed in the tumors seen in 20 NSCLC patients. The authors noticed that inter-scan variability of the features varied depending on the feature itself and the cartridge material. One of the drawbacks of the CCR phantom is its uniform cartridge shape, which cannot study radiomics features that describe tumor shape and the interrelation between tumors and surrounding tissue.

Other studies also used the CCR phantom to explore the reproducibility and robustness of radiomics features across CT scanners, scanning techniques, and reconstruction parameters. An example was to study the effect of CT tube current on radiomics features. Using the ten cartridges in the CCR phantom, one study showed no clear effect of tube current on radiomics features (33). Another study, however, showed that tube current affected features extracted from homogeneous materials more than from tumor-like textured phantoms when splitting 6 cartridges contained in the CCR phantom into two groups, one filled with homogeneous materials and the other filled with more tissue-like texture materials (40).

#### 3D Printed Phantoms

Although the CCR phantom has been widely used to investigate variability in radiomics features across scanners and scanning parameters, it does not contain lesion shape information, and its density textures/patterns are not anatomically informed. Recently, advances in 3D printing technology have made it possible to design and fabricate synthetic phantoms with realistic lesion sizes, shapes, intensities and internal textures while knowing the ground truth of their characteristics.

Using a subset of lung nodules taken from the database of Lung Image Database Consortium (LIDC), a series of corresponding virtual nodule models were created using the investigators’ software and its built-in fitting and texture modeling routines (63). A multi-material 3D printer then distributed 2 base materials in the desired proportions according to the dithered nodule model to achieve lesion sizes, shapes, and internal density textures similar to those of the real nodules. The heterogeneous nodule phantoms were imbedded in an anthropomorphic thoracic phantom and scanned using different acquisition parameters of dose level, slice thickness, and reconstruction kernel. The study demonstrated that the printed textured phantoms can be used to determine the variability and accuracy of texture features extracted from CT images acquired at varying imaging settings.

In order to determine robust shape features, researchers used spherical harmonic functions to create mathematical tumor models with increasing degrees of complexity/spiculatedness and printed the models using a single material 3-D printer (64). They studied the relationship of a set of commonly used shape features (e.g., Volume, Surface area, Compactness, Sphericity) with varying degrees of spiculatedness under different conditions (slice thickness, resampling, and surface and volume computing algorithms). As expected, they found that surface-specific features, such as Surface area, were positively correlated with tumor spiculatedness, whereas global shape features, such as Compactness, were negatively correlated with tumor spiculatedness. They also found that the shape features are less affected by the aforementioned variables and less dependent to tumor volume.

### Efforts Made in Imaging Harmonization

Image acquisition settings can vary considerably in datasets collected from retrospective or ongoing multi-center studies. Radiomics signatures that are influenced by variations in the source imaging settings may assign significance to differences such as an imaging parameter used to reconstruct images, rather than the biologically significant differences in tumor images. Establishing the consistency of image data is vitally important for the discovery of robust imaging biomarkers which can be validated and applied to multi-center clinical trials and clinical practice. Harmonization of imaging protocols is an effective approach to reduce imaging-induced variability in radiomics.

#### Identifying Comparable Imaging Parameters

Different imaging settings can be said to be comparable when similar feature values can be computed from the images they produce. An early effort to identify comparable imaging settings was reported by the team who had contributed the RIDER Lung dataset. As a subsequent analysis of the same-day repeat CT study, the investigators used the six-setting CT image data to assess the feature agreements across the 3 slice thicknesses and 2 reconstruction kernels (45). Three inter-setting comparisons, i.e., 1.25S vs 2.5S, 1.25L vs 2.5L and 2.5S vs 5S, show high average CCC values (> 0.8 for all feature groups; bottom row in **Figure 4B**), indicating that these imaging parameters can be used interchangeably in radiomics studies. The study also found that changing slice thickness alone can generate better agreements, especially when the range of slice thickness is limited to 1.25mm and 2.5mm. Furthermore, combining thicker slices with sharper reconstruction algorithms can have the same effects as combining thinner slices with smoother reconstruction algorithms for the computation of radiomics features.

#### Controlling Imaging Protocols

The team who developed the CCR phantom studied whether a controlled imaging protocol could reduce variability in radiomics features (35) by scanning an updated version of the CCR phantom on 100 scanners using both local and study-specified CT protocols for chest and head & neck (H&N). The local imaging protocols were heterogeneous, e.g., the slice thickness ranged from 1 mm to 5 mm, while the study-specified protocols were controlled by using comparable imaging parameters across scanners, e.g., the reconstruction used slice thicknesses of 2.5 mm or 3 mm and smooth kernels. The size of cylindrical ROIs was 8.2 cm in diameter. The IBEX radiomics package was used to calculate 49 features including Neighborhood Grey Tone Difference Matrix (NGTDM) and Grey Level Co-Occurrence Matrix (GLCM). A linear mixed effects model was used to determine the overall variability contributed by the manufacturer, scanner of a given manufacturer, cartridge material, and residual to the variability in the measurements. The authors found that, compared to the local chest and H&N imaging protocols, the controlled protocols could reduce the overall variability by 57% and 52%, respectively.

Optimal standardization of chest imaging protocol parameters did not ensure the reproducibility of 27 texture features from the NGTDM and GLCM families, which were also computed using the IBEX radiomics package, across three CT vendor scanners (34) in a study using an anthropomorphic lung phantom with inserted lesions of different materials that simulated the attenuation properties of a human tissue. The imaging parameters were optimally chosen for lung cancer studies except the reconstruction slice thickness of 5 mm, which was rather thick for the small phantom lesions that ranged from 1 cm - 1.5 cm. One limitation of this study, as discussed by the authors, was the small size of the lesion inserts. The authors planned to conduct a follow up study to investigate the impact of ROI size on feature reproducibility, as calculating texture features such as GLCM from relatively small lesions on thick slice thickness can be problematic.

It is hard to make a direct comparison between the findings of these two phantom studies exploring benefits of the imaging parameter harmonization across CT scanners due to the differences of the phantoms, image preprocessing, etc.

#### Converting Imaging Settings to Desired Setting

Artificial intelligence (AI) offers the potential to automatically harmonize images which were acquired and reconstructed at different imaging settings. A recent study reported the use of a CNN to improve the reproducibility of radiomics features between different reconstruction kernels (soft and sharp) (65). The investigators developed a CNN architecture using residual learning and an end-to-end approach. To demonstrate the effectiveness of this CNN model, a total of 702 radiomics features were extracted from 104 pulmonary nodules or masses (all >= 6 mm; 51 non-enhanced and 53 enhanced CTs) using Pyradiomics (66), an open-source feature extraction package. The CCCs of the total features extracted from images reconstructed at the different kernels and the different kernels after image conversion were 0.38 and 0.84, respectively. Among the features, the CCCs of the wavelet features increased the most after the image conversion of the reconstruction kernels. The authors concluded that CNN-based CT image conversion can reduce the effect of reconstruction kernels on radiomics features. Another study showed that CNN-based super-resolution methods can improve the reproducibility of radiomics features extracted from CT images reconstructed at different slice thicknesses (67).

#### Matching Image Appearance/Quality

Differences in image quality between special vendors’ CT systems are unavoidable. In addition to the scanner equipment, tube voltage and current, FOV, slice thickness, and reconstruction kernels, there are many other acquisition-related “hidden” factors that may affect image quality. It is impossible to study all affecting factors, known or unknown, one by one.

An alternative way to reduce feature variability caused by imaging is to identify the similarity of images acquired at different settings. Phantom studies can help match image appearance and thus identify comparable imaging settings across different vendors’ scanners, scanning techniques and parameters, etc. (68, 69). For example, by analyzing noise power spectrum (NPS), a group of medical physicists conducted a study using the ACR CT phantom to quantitatively compare noise texture between two CT systems, GE and Siemens (68). Under a consistent acquisition protocol (120 kVp, 0.625⁄0.6 mm slice thickness, 250 mAs, and 22 cm field of view), using filtered back projection and a wide selection of available reconstruction kernels, a systematic kernel-by-kernel comparison was performed. The study found that the GE’s “Soft,” “Standard,” “Chest,” and “Lung” kernels closely matched the Siemens’ “B35f,” “B43f,” “B41f,” and “B80f” kernels, respectively. More research in matching image quality can be found in (69).

#### Identifying Images Acquired at Optimal Phase Timing

Multi-phase CT scans after contrast administration are most widely used for liver cancer diagnosis, prognosis, and response assessment. Bolus tracking is used clinically during image acquisition to control (arterial and portal venous) phase timing to increase the likelihood of the phase timing being optimal. However, bolus tracking does not consider individual patient’s biological variation and thus cannot ensure that the optimal timing was successfully reached in a given patient. In a pilot study, investigators explored the effect of portal venous phase (PVP) timing on the density measurement of liver metastases (LM) from colorectal cancer (CRC) and found that LM-CRC density was significantly decreased at non-optimal PVP timing by 14.8%: 16.7% at early PVP and 12.6% at late PVP (49). The same group then developed both semi-automated and AI-based fully-automated programs to identify optimal from non-optimal PVP timing as well as to differentiate five contrast-enhancement phases (49, 70, 71). They applied the developed PVP optimal-timing quality assurance (QA) method to their study developing an on-treatment signature to detect metastatic CRC patients sensitive to FOLFIRI+cetuximab using radiomics analysis of tumor changes between baseline and 8-week CT images. The radiomics signature showed higher performance on optimal imaging (AUC=0.80; 95%CI:0.69, 0.94) than on non-optimal imaging (AUC=0.72; 95%CI:0.59, 0.83) (72).

The effect of optimal timing on radiomics features is an understudied area. Automated AI-based QA algorithms to identify optimally acquired CT scan images for radiomics analyses can help ensure image quality and consistency and thus increase the chances to develop reproducible and reliable radiomics signatures.

### Influence of Imaging Harmonization and Optimization on Radiomics Models

Imaging harmonization has shown potential for improving the reproducibility of radiomics features. The following subsections review and discuss how the performance of predictive models built using radiomics features is affected by the harmonization and optimization of image acquisition parameters.

#### Diagnosis of Solitary Pulmonary Nodule (SPN)

In a study using radiomics signatures to help the diagnosis of SPN, investigators assessed the effects of contrast enhancement, slice thickness, and reconstruction kernel on the diagnostic performance of the model they developed (73). In total, 240 SPN patients (malignant:benign = 180:60) had both non-contrast CT (NECT) and contrast-enhanced CT (CECT) scans, each reconstructed using two different slice thicknesses of 1.25 mm and 5 mm and two reconstruction kernels of lung (sharp kernel) and standard (smooth kernel). At each CT imaging setting, 150 radiomics features were extracted from each SPN and the diagnostic performance of the resulting signature was assessed based on its AUC. The validation results showed better discrimination capability of the radiomics signature derived from NECT than CECT (AUC: 0.750 vs. 0.735, p=0.014), from thin-slice than thick-slice CT (AUC: 0.750 vs. 0.725, p = 0.025), and from smooth kernel than sharp kernel (AUC: 0.725 vs.0.686, p = 0.039). The authors thus concluded that the non-contrast, thin-slice (1.25mm) and smooth reconstruction kernel-based CT was more informative for SPN diagnosis compared to the other imaging parameters studied.

#### Prediction of EGFR Mutation Status in Lung Adenocarcinoma (LAC)

Investigators evaluated whether the optimal selection of CT reconstruction settings improved the construction of a radiomics model to predict EGFR mutation status in LAC using standard of care CT images (74). In this study, CT scans of 51 patients (EGFR : WT = 23:28) with LACs of clinical stage I/II/IIIA were reconstructed at the following four image setting groups: 1) Thin-Sharp, 2) Thin-Smooth, 3) Thick-Sharp, and 4) Thick-Smooth (Thin: 1 mm; Thick: 5 mm; Sharp: B70f/B70s; Smooth: B30f/B31f/B31s). In total, 1,160 radiomics features were extracted and used to build machine learning prediction models at each of the four settings and a mixture setting (cases randomly selected from the groups 1-4). The study showed the best AUC (95%CI) of 0.83 (0.68, 0.92) when using the Thin-Smooth setting and the worst AUC (95%CI) of 0.75 (0.59, 0.86) when using the mixture setting (P<10^-3^).

#### Prediction of Overall Survival (OS) in Head and Neck Cancer

A recent radiomics study in head and neck cancer found that models built with patients on a controlled imaging protocol did not predict OS better than models built using varying imaging protocols (75). In this study, investigators retrospectively collected 726 patients’ CT images from one U.S. and two European institutions, among which the largest subset of 511 patients’ CT images was acquired using a GE scanner with the reconstruction parameters of a standard kernel and 1.25 mm image thickness. The radiomics features were computed using IBEX (62). Radiomics models to predict OS were built using the full patient dataset (heterogeneous imaging protocols) and the largest subset (controlled imaging protocol). This study did not find increased performance of the outcome prediction model when the imaging protocol was controlled (AUCs: full set vs. subset = 0.72 vs. 0.55). Moreover, volume and HPV status were selected as covariates in the OS prediction model built on the full patient dataset. The authors further reported that volume alone or volume and HPV status provided an AUC of 0.73, indicating that adding radiomics features did not improve the model performance. This again suggests that radiomics texture features can be a surrogate for/correlated with tumor volume and points to the need to remove redundant features prior to model building (3, 76).

### Discussion: Potential Strategies and Practical Considerations to Reduce Variability in Image Acquisition

Image acquisition is the first essential step in the radiomics workflow, directly determining the quality of images upon which all subsequent analyses rely. Some strategies and considerations to improve image consistency and reduce feature variability are highlighted below.

*Controlling Imaging Protocols To Increase Image Consistency*. CT scanners, scanning techniques, and reconstruction parameters can affect radiomics features and models. The degree of variation caused by these factors depends on the tumor’s characteristics and the radiomics feature itself. Studies should report imaging acquisition settings in detail so that they can be reproduced by others. Ensuring high quality and consistent images across scanner devices and imaging protocols is the key for the successful development and application of radiomics signatures.

*Potential Optimal Imaging Parameters For Studying Lung Cancer Phenotypes*. Controlling CT imaging protocols and complying with these protocols are essential to the acquisition of high quality and consistent image data for radiomics studies. Preliminary data suggest that the most suitable imaging parameter setting for phenotype studies in lung cancer is thin slice thicknesses (e.g., 1 mm, 1.25 mm) and smooth reconstruction kernels (e.g., standard, B31f/B31s). Moreover, same-day repeat CT studies found that the settings of 1.25S and 2.5S generated the most reproducible features (**Figures 4A, B**). These independent findings support the use of thinner slice thickness and smoother kernel for prospective lung cancer phenotype studies. However, this approach warrants further investigation, especially because of the conflicting findings in the H&N study.

*Test-Retest With Proper Imaging Parameters*. The purpose of test-retest is to identify radiomics features that are sensitive to re-imaging and remove them from subsequent analyses. Because image acquisition parameters can affect computed feature values, the imaging parameters should be matched, or adequately similar, between the test-retest data and the individual studies’ data so that the testing results are reliable. In addition, different disease sites should have their own test-retest image data, which can be acquired from either patients or (texture) phantoms. Before re-shooting a phantom, make sure to relocate/re-orient the phantom. When test-retest imaging is not available for the phenotype of interest, image perturbation such as noise addition, image translation and rotation, and volume growth or shrinkage can be considered (77).

*4D CT - An Alternative For Test-Retest*. Scanning patients twice during a short time period is impractical. However, 4D CT imaging has been used in radiotherapy to reduce respiratory motion-induced changes in tumor location and morphology. Such image datasets are often available in radiation oncology departments. Due to its ability to generate 3D CT image series at multiple respiratory phases, the 4D CT scan images can serve as a candidate of test-retest dataset to investigate feature variability. Studies show that certain radiomics features are sensitive to respiration and the preselection of 4D stability features can improve the performance of radiomics prediction models. Moreover, the end of the exhale phase, which is less affected by respiratory motion compared to the other phase images, is recommended to reduce feature variability for radiomics studies.

*Radiomics Phantoms*. Phantom studies play an essential role in exploring different sources of variation and their magnitudes across vendor scanners, scanning techniques and reconstruction parameters. However, traditional physical phantoms are usually constructed of materials that are radiologically equivalent to tissues and contain simple geometric features such as cartridges, cylinders, line-pair patterns, and ramps. Anthropomorphic phantoms typically mimic the overall shape of a human being but don’t include detailed intra-organ/lesion features and are mostly used for dosimetry measurements. Thus, there is a significant gap between the intricate anatomical details that are seen in clinical CT images and the mostly uniform and simple nature of traditional physical phantoms. Characterizing such synthetic lesions using the cutting-edge 3D printing technology would be instrumental toward assessing the variability of features across different CT platforms and protocols.

*Quantitative Metrics To Determine Image Quality and/or Similarity*. The wide range of vendors’ scanners, scanning techniques, and reconstruction parameters used in clinical practice and clinical trials makes it impossible to study the effects of all possible variables on radiomics features and models. Developing quantitative methods/metrics to determine image quality and/or similarity can be an alternative way to identify comparable images that can be used interchangeably or to decide whether an image’s quality is adequate for computing radiomics features. This should be done based on the acquired images such as identifying optimal phase-timing, with no need to know the exact acquisition parameters of the images.

*Imaging Harmonization Through AI/CNN*. Image processing methods can reduce variability in images acquired with heterogeneous image acquisition settings. Voxel size resampling followed by Butterworth smoothing (an image processing method) has been found to improve feature reproducibility (42). Traditional image processing methods cannot be automatically adapted to harmonize a multitude of imaging settings that could exist in an image dataset. AI/CNN, however, shows great promise in converting CT imaging settings to a desired setting and in identifying whether images are acquired at the optimal phase timing. There is no doubt that AI, especially generative adversarial network (GAN)-based networks, will play a significant role in image-to-image translation including CT imaging conversion/harmonization (78).

*Reproducible Features vs. Clinically Informative Features*. When investigators report the improved reproducibility of radiomics features, a common method is to count the increased number of the studied features that have increased CCC values or CCC values greater than a predefined threshold (e.g., CCC >0.85). It is true that when more features are reproducible, there is a greater likelihood to identify robust radiomics models which are built using these reproducible features. However, the reproducibility of a feature does not necessarily mean that it is clinically informative. On the other hand, it is likely that heterogeneous imaging settings may have little effects on some coarse clinically informative radiomics signatures such as ground glass opacity (GGO) portion and necrosis component of a tumor. Nevertheless, successful radiomics models must be built upon reproducible and robust features.

## Lesion Segmentation

Lesion segmentation is a prerequisite for feature extraction, a critical step in radiomics workflow. Segmentation is an essential part of computer vision and image processing and is still an active research area today. Artificial intelligence (AI) promises fully-automated detection and segmentation of lesions (79).

### Segmentation Methods and Variability

Segmentation can be performed manually, semi-automatically, or fully-automatically. Variability of the lesion segmentation may come from diverse segmentation algorithms and human supervised post-segmentation correction.

#### Manual Segmentation

Manual segmentation, a hand-drawing method using a computer mouse, is used only when there is no access to reliable semi-automated segmentation software because it is time consuming, subjective, and prone to variability due to radiologists’ different opinions on identifying lesion boundaries (inter-reader variability) or a radiologist’s inconsistency in delineating lesion boundaries at different time points (intra-reader variability). Manual segmentation was still used in about 40% of the lung cancer radiomics studies in our literature search (**Table 1** in **Supplemental Materials**).

#### Semi-Automated Segmentation

Semi-automated segmentation requires an operator to use a computer mouse to manually initiate a segmentation algorithm that can be developed using different strategies such as clustering, region-growing, active contours, and watershed transform.

##### Inter-Algorithm Variability

**Figure 5:**
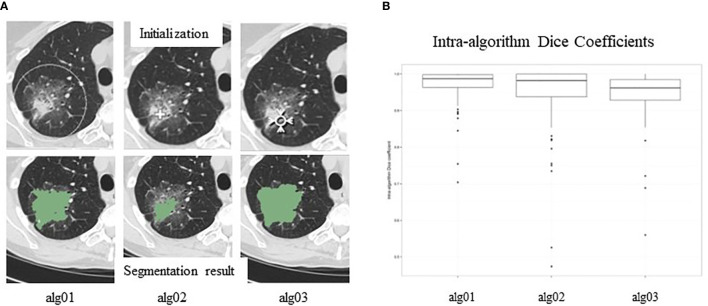
**(A)**. Inter-reader variability in segmentation. Top panel: manual initializations (seed point/ROIs) of three segmentation algorithms; bottom panel: corresponding segmentation results. **(B)** Intra-reader variability in segmentation. Segmentation results are affected by seed points/ROIs. Reproduced with permission from (80).

Different strategies employed in different segmentation algorithms can yield different results (inter-algorithm variability). In a “moist run” dataset (40 lung lesions and 12 lung phantom lesions) collected by the Quantitative Imaging Network (QIN) for a lung segmentation challenge, large variations were seen when three different segmentation algorithms were applied to the same GGO lung lesion (**Figure 5A**) (80). Briefly, the algorithm Alg01 was based on the marker-controlled watershed transform and required a region-of-interest (ROI) manually drawn outside the lesion as the algorithm’s initial input (**Figure 5A**, top-left). Alg02 and Alg03 used the region-growing approach, with either one or multiple clicks to determine seed points (Alg02) (**Figure 5A**, top-middle) or a seed circle (Alg03) (**Figure 5A**, top-right) inside the lesion as the initial input. For heterogeneous lesions, the region-growing based algorithms can easily be trapped by a local homogeneous region, creating a high risk of under-segmentation. In this example, Alg02 segmented only the solid part of the lesion (under-segmentation) when the seed point was placed in a high-density area of the lesion.

##### Intra-Algorithm Variability

After manual initialization, each algorithm analyzes the density distribution of the pixels provided by the initial ROI and then automatically separates the lesion from its background using the input information and its own segmentation strategy/objective criteria. Therefore, initial ROIs can affect segmentation results. **Figure 5B** shows variations (Dice Coefficient distribution) of the segmentation results (lesion volumes) of each of the three algorithms when initial ROIs are placed differently. This kind of variation is called intra-algorithm variability. A good segmentation algorithm should be insensitive to initial ROIs. Studies reported that radiomics features extracted from segmented lesions had higher reproducibility when using the same algorithm with different initial inputs than when using different segmentation algorithms (81, 82).

#### Fully-Automated Segmentation

Fully-automated segmentation is performed without any human-machine interaction. The input of such algorithms is the entire image series and the output is the image series containing automatically segmented lesions. A fully-automated segmentation method needs to perform lesion detection and segmentation simultaneously. The challenge for automated lesion detection is to avoid false negative and false positive lesions. Unlike manual and semi-automated segmentations, repeatedly running a fully-automated algorithm on one image series won’t change the output result. However, the impact of image acquisition settings on fully-automated segmentation algorithms needs to be explored (83).

### Human Supervised Post-Segmentation Correction

Ideally, a lesion segmentation algorithm should be fully automated, reproducible, and accurate. However, both lesions and relationships between lesions and their surrounding tissues can manifest in complex patterns on CT, making a satisfactory segmentation for all lesions unrealistic. To avoid segmentation errors, a radiologist needs to review and correct computer-generated lesion contours. Over the past few years, awareness of the need for human supervised post-segmentation correction has increased (**Figure 2B**; green color). Supervised segmentation is influenced by the radiologist’s subjective judgement. However, only the modified parts of the lesion contours are affected by the manual correction and the unmodified contour parts are still determined by objective criteria. This explains why radiomics features extracted from lesions segmented manually were less reproducible than those extracted from lesions segmented algorithmically with supervision by a radiologist (84, 85).

### Segmentation of Multiple Disease Sites

Solid tumors, including primary and metastatic lesions, exist in various organs. They can present various intra-tumoral patterns and contrast levels to the surrounding tissues on CT images, which challenges lesion segmentation to different degrees. For instance, lung lesions are usually easy to be segmented due to their high contrast to the surrounding lung parenchyma. However, when lung lesions attach to blood vessels or chest walls possessing similar densities to those of the lesions, segmentations can become difficult. Lymph nodes are well-known for their low contrast to their surrounding backgrounds. Segmentation of liver lesions can suffer from their heterogeneity, low contrast against liver parenchyma (contrast-enhancement dependent), and noisy abdominal images. Various strategies have been developed to better delineate tumors of different types.

In general, texture features are affected more than volume feature by image acquisition parameters. Over-segmentation, i.e., inclusion of surrounding non-lesion tissues in the lesion segmentation, can have a large effect on texture features when there is a large density difference between the lesion and its surrounding tissues (e.g., lung lesion and lung parenchyma). A tight segmentation is thus more desired than a loose segmentation in radiomics studies. Lesion segmentation can hit lesion boundary-related features harder than others.

A study preliminarily analyzed the effect of inter-observer variability between three manual contours on the stability of 1,404 radiomics features in head and neck squamous cell carcinoma (HNSCC), malignant pleural mesothelioma (MPM), and NSCLC (86). There were 11 lesions for each type. The authors found that the inter-observer delineation variability was the highest in MPM and the lowest in NSCLC, and the stability rate of radiomics features negatively correlated with delineation variability. Shape-related features showed the weakest stability among the 3 tumor types.

### Effect of Inter-Reader Variability on Radiomics Prediction Model

The last example in this section shows a pilot study exploring the effects of inter-reader variability on radiomics prediction models. In the study, the investigators predicted EGFR mutational status in early stage NSCLC patients treated with a targeted therapy (Gefitinib) using the change in 89 radiomics features over 3 weeks (delta features) extracted from 1.25 mm and lung kernel images (87). Lung lesions in 46 patients (EGFR:wildtype = 20:26) were independently segmented by three radiologists using in-house software that allowed manual post-segmentation correction. Univariate analysis identified the most significant delta features computed from each of the three radiologists’ segmentation results. The best EGFR prediction performance expressed by AUC values differed for each radiologist's segmentation: 0.79 (top feature: compact factor – a shape feature), 0.85 (top feature: mean density) and 0.91 (top feature: volume), respectively. Delta volume was the only feature that was among the top 5 most significant features in all three radiologists’ results. The prediction performances using the delta volumes obtained by the three radiologists were (AUC=) 0.77, 0.80 and 0.91, respectively. All outperformed the corresponding unidimensional performances of 0.63, 0.53 and 0.66. Unidimensional measurement (i.e., tumor in-plane diameter) is used to assess tumor change by conventional Response Evaluation Criteria in Solid Tumors (RECIST) (88). None of the three radiologists’ results included the delta diameter in its top 5 most significant feature list. The results of this study warrant validation on larger data.

### Open Source Software for Lesion Segmentation

3D Slicer and ITK-Snap are the most popular open source platforms for interactive segmentation, registration, and volume rendering/visualization of medical images. Built over two decades through support from the National Institutes of Health (NIH) and software engineers worldwide, 3D Slicer has provided researchers with a set of free image processing tools (89). ITK-SNAP is another open source tool that offers free semi-automatic segmentation software (90). Both platforms provide manual delineation functions. So far, about 25% of lung cancer radiomics studies were conducted with the help of open source segmentation tools (**Table 1** in **Supplemental Materials**).

### Discussion: Potential Strategies and Practical Considerations to Reduce Variability in Lesion Segmentation

Accurate, reproducible, and efficient segmentation tools that can be widely distributed are essential to accelerating and advancing cancer imaging research. Semi-automated segmentation tools have commonly been used in radiology-oncology imaging studies. An imaging platform providing lesion segmentation software should also provide a manual editing/correction function. Certainly, computer segmentation methods are more efficient when they require fewer human-machine interactions.

*Inter- and/or Intra-Reader Test*. The Purpose of Inter-Reader (or intra-reader) testing is to recognize radiomics features that are sensitive to lesion segmentation so that they can be removed from subsequent analyses. Features that are sensitive to segmentation can be identified by asking multiple radiologists to delineate the same lesions or an individual radiologist to delineate a set of lesions at two or more sessions, with a sufficient time interval between any two annotation sessions to avoid the effects of the radiologist’s reading memory.

*Radiologists’ Consensus on Lesion Contouring*. Radiologists are not specifically trained in identifying tumor boundaries; big variations can happen especially when segmenting partial solid tumors. The Tumor Segmentation step shown in **Figure 1** (Radiomics workflow) offers an example of three radiologists’ manually delineated tumor contours; some tend to delineate contours tightly surrounding a solid tumor component, while others tend to delineate contours loosely including more GGO areas. Although there may not be “gold standard” lesion boundaries, obtaining radiologists’ consensus about lesion boundaries can help reduce variability in segmentation and thus in computed radiomics features.

*Proper Use of Segmentation Software*. Different semi-automated algorithms use different strategies to obtain information about lesions and/or their surrounding tissue from initial ROIs, which can help the algorithms identify lesion pixels/voxels. For instance, to properly start a region-growing based algorithm, seed ROI points/circle should be placed in both hypo and hyper density areas inside a heterogeneous lesion so that the range of lesion densities can be fully captured and used to guide the region growing algorithm. Proper use of a segmentation algorithm can improve the segmentation’s accuracy and consistency. Again, to avoid unpredictable surrounding tissues of possible high (or low) contrasts, tight segmentation results are more preferred than loose segmentation results in radiomics studies.

In radiation oncology, the standard treatment planning process has generated a large amount of annotated tumors that can be readily used in radiomics studies. However, it should be noted that the quality of the segmentations might not be sufficiently precise for radiomics. For instance, there is no need to accurately delineate the speculated edges of a tumor for the purposes of treatment planning while radiomics requires a very precise delineation of the tumor. Therefore, segmentation results taken from radiotherapy data may need to be refined prior to feature extraction.

*Effects of Imaging on Segmentation*. Acquisition settings determine image quality and can thus affect segmentation algorithms [e.g., (91, 92)]. The ultimate goal of image pre-processing is to reduce noise while maintaining image details. Generally, pre-processing methods using smoothing filters (e.g., Gaussian filter) are applied for the region-growing based algorithms, whereas sharpening filters (e.g., Laplacian filter) are used by the edge-based segmentation algorithms. When investigating volumetric imaging biomarkers, variables affecting volumetry/tumor segmentation have been intensively studied, particularly by the RSNA-organized Quantitative Imaging Biomarkers Alliance (QIBA) (93–95), which is not further discussed in this review.

## Feature Extraction

Radiomics features are also known as quantitative image features. In the past decades, pattern recognition using quantitative image features has been widely used for tasks such as image segmentation, classification, and computer-aided detection and diagnosis (96).

### Radiomics Features

Radiomics features can be grouped into two categories: agnostic and quantified semantic features (18). Agnostic features are derived to quantify lesion morphology and density heterogeneity through mathematical equations/descriptors, while quantified semantic features are developed to characterize visual patterns of lesions (ROIs) based on radiology lexicons. Agnostic features are usually further divided into the following four categories based on: 1) morphology (e.g., size, shape), 2) histogram-statistics (e.g., mean, standard deviation, skewness, kurtosis), 3) texture (e.g., Run-Length, GLCM), and 4) transformation (e.g., Wavelet transform). Histogram-based features, a.k.a. first-order statistics, describe tumor density distribution without considering spatial information, whereas texture features, a.k.a. second-order statistics, characterize tumor heterogeneity by considering the spatial interrelations of image pixel/voxel densities. Transformation-based features are computed from transformed images rather than original images. Of note, there is another type of quantitative features that can provide additional information, i.e., features that characterize density transition between a lesion and its surrounding tissues/parenchyma. An example is the feature class of Sigmoid Function; the feature, Sigmoid-slope, can be used to quantify lesion edge (density) sharpness.

Quantified semantic features are perceptive because they are created based on a radiologist’s visual observations. For instance, GGO volume percentage, a quantified visual feature, was found to be significantly higher in tumors with exon 21 missense mutation than that in tumors with other EGFR mutation status (97). Some agnostic features can also be intuitively interpreted. Skewness, an agnostic feature that measures the asymmetry of a density distribution about its mean (e.g., density distribution of a solid tumor is left-skewed with a negative skewness value), was found to be predictive for disease-free-survival (DFS) associated with certain histologic subgroups of lung adenocarcinoma; the lower the skewness value is, the poorer the DFS will be (98). Another example is the Laws’ Energy features. This feature class emphasizes texture patterns of edge, spot, ripple and wave through the Laws filters. Whether such tumor image patterns are clinically informative needs to be investigated. However, meanings of many agnostic features can be hard to be intuitively interpreted. Nevertheless, it is believed that models built upon one or multiple radiomics features can distinguish imaging phenotypes that can or cannot be visually observed by human.

### Sources of Variation in Feature Computation

Traditional radiomics features are computed from predefined mathematical equations/descriptors that can be found in textbooks and/or published literature (99). Theoretically, these radiomics features are clearly defined and thus fully controllable. However, sometimes there are multiple choices to define a feature with an identical name, select specific values for feature parameters, and implement a feature calculation. In reality, values of radiomics features computed using different feature extraction software can vary considerably, which makes it hard to compare radiomics studies especially if details of the feature definitions, parameter settings, and implementations are not disclosed adequately.

#### Feature Definition

Variations in feature definition can happen when multiple equations/descriptors are used to define a same feature. A simple example is Compactness, a shape feature that is defined to quantify how spherical a 3D object’s shape is. Although Compactness is a function of an object’s surface area (S) and volume (V), there are different equations to define it, e.g., V/(π ^½^_*_ S ^3/2^) and 36_*_π_*_ V^2^/S^3^. Even if these two equations are related, the computed values from the two equations are different. This type of variance can be controlled by making feature definitions transparent.

#### Feature Parameter Setting

Many features, especially texture features, have parameters in their definitions so that they can be used to quantify image patterns at multiple scales and different orientations. The feature parameter used most often is the number of gray-level (density) bins, a.k.a. the bin level. Density discretization groups the entire density range of images into bins of equal width. Reducing the bin level or increasing the bin width can improve the computational efficiency for certain features such as GLCM features. Moreover, density discretization can lessen noise interference. In general, bin width should not be lower than random noise level. However, a large bin width may not be able to capture the subtle differences in density (texture).

**Figure 6:**
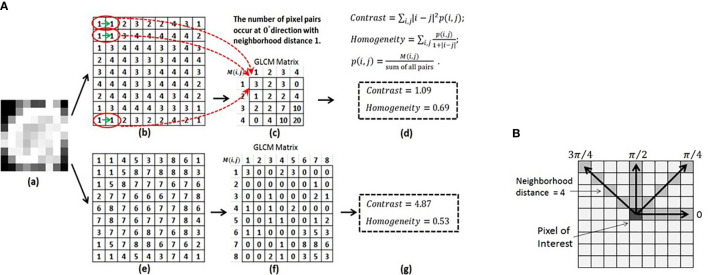
**(A)** Computing the GLCM features of Contrast and Homogeneity using different bin levels. (a) Original image. (b) Normalized image using the bin level of 4. (c) GLCM matrix derived from (b). (d) Contrast and Homogeneity computed from GLCM in (c). (e) Normalized image using the bin level of 8. (f) GLCM matrix derived from (e). (g) Contrast and Homogeneity computed from GLCM in (f). **(B)** Calculation of the GLCM features for a 9X9 2D image at four directions and a neighborhood distance of 4 pixels. Reproduced with permission from (100).

The GLCM feature class is an excellent example to explain feature specific parameters (**Figure 6**) (100). A GLCM matrix is created by counting how often pairs of pixels with specific gray-level values occur in a specified distance and direction over the ROI. The GLCM features are the computed statistics from the matrix (101). There are 3 key parameters: the bin level of the original images (i.e., the dimension of GLCM), the distance of pixel pairs, and the direction of the line spanned by the pixel pair. In **Figure 6A**, starting with an original image, the figures show the feature computation process. Two example GLCM matrices are generated with the bin levels of 4 [**Figures 6A(b)–(d)**] and 8 [**Figures 6A(e)–(g)**], both with the distance of 1 pixel and direction of 0° (**Figure 6B**). For each GLCM matrix, two common GLCM features, Contrast and Homogeneity, are calculated and their values are different due to the different bin levels, **Figures 6A(d)** and **(g)**.

The influence of density discretization (bin levels) on radiomics features was investigated using the CCR phantom (33). The effect of the bin width (5 to 50 HU) on the stability of 114 studied texture features was found to be marginal compared to the effect of scanners, slice thicknesses, and tube currents. Although the study concludes that feature stability may not be compromised during the optimization of gray-level discretization when attempting to improve model performance, evidence from clinical studies is needed.

#### Feature Implementation

Often, there are multiple choices to implement certain radiomics features. For instance, a lesion surface area can be evaluated by a mesh-based representation of the outer surface or by areas of voxel faces toward the outside of the lesion. For features that are derived from pre-processed images using a filtering technique such as Gabor filter, filter length is a feature parameter, and the method for handling the ROI edge when moving the filter over the ROI is a hidden variable in the implementation of the Gabor filter. Moreover, features can be computed in 2D, 2.5D (a combination of 2D features), or 3D and extracted from the original images as well as from pre-processed images using different filtering techniques. In 2D image processing, for instance, 4 or 8 connected pixels are usually considered as neighboring pixels and 4 or 8 directions are chosen. All these and more unspecified variances during feature computation/implementation can add unknown variation to the computed feature values. To date, no radiomics studies have provided sufficient details about their feature definitions, parameter settings and implementations so that others can reproduce this aspect of their studies.

### Studies Exploring and Reducing Variability in Feature Computation

Research has investigated sources of variation in feature computation (81, 102–104). Two collaborative studies on this topic are reviewed and discussed in the following subsections.

#### Preliminary Effort by the Quantitative Imaging Network (QIN)

This study, conducted by ten teams from the PET/CT working group of the QIN funded by the National Cancer Institute (NCI), explored the agreement of 13 software packages on nine basic radiomics features including volume, 2D and 3D diameters, mean density, standard deviation, kurtosis, surface area, sphericity, and GLCM entropy (103). The investigators applied the feature extraction software used by the teams (about half open source and half in-house) to both Digital Reference Objects (DROs) and patient image data. The DROs consisted of three objects with both texture and uniform densities and spherical and spiculated shapes (105). The patient data contained images from 10 patients taken from the LIDC database, a publicly accessible database (106). One pre-annotated contour for each DRO/lesion was used to extract radiomics features. Percentage coefficient of variation (CV) was used to evaluate agreement of the computed features. The results showed that for the DROs, six out of the nine features, i.e., volume, 2D and 3D diameters, mean density, standard deviation and kurtosis (after Fisher correction), demonstrated excellent agreement (CV < 1%). The features of surface and sphericity showed moderate agreement (CV: ~13%). GLCM entropy had big variations (texture DRO: ~50%; uniform DRO: CV > 600%). For the patient data, CV values of 2D and 3D diameters, surface, and sphericity increased but were still moderate. CV of the GLCM entropy decreased to ~36%. All other features remained in excellent agreement.

From the DROs to real lesions, ROI shapes became more irregular and densities became less uniform. This was why the software packages turned out to agree less with each other when computing features that relied more on ROI boundaries/surfaces, such as 2D and 3D diameters and surface and sphericity features. It was not surprising that the GLCM entropy feature showed such big variations between feature extraction software packages. Harmonization of some key parameters (e.g., bin level, pixel pair’s distance and direction) was found to reduce the average CV value of GLCM entropy from ~36% to ~20%.

#### Comprehensive Study by the Image Biomarker Standardization Initiative (IBSI)

Since 2016, the IBSI, an independent international collaboration, has focused on standardizing definition and implementation of quantitative image features and providing benchmark data sets and consensus-based reference values (26). The IBSI reference manual is written to provide consensus-based recommendations and guidelines to improve reproducibility and transparency of radiomics features and studies.

Recently, the IBSI published a large scale study that standardized 169 commonly used radiomics features (104). This multi-year, multi-phase study involved 25 research teams using their own feature extraction and image processing software and showed the investigators’ first-hand experience in the calibration and certification of various feature extraction software packages. The study utilized a consensus-based and iterative approach. Phase I (25 participating teams) obtained the reference values of radiomics features based on a 3D digital phantom. Phase II (15 teams) defined a general image processing scheme, implemented it at different configurations, and obtained corresponding reference values of radiomics features using a lung cancer CT image series. Initially, only weak consensus (<3 teams matched) existed for 76.8% features at phase I and 65.4% features at phase II. At the final iteration, strong or better consensus (6-9 team matches) was achieved to 95.1% and 90.6% at phase I and II, respectively. Phase III (9 teams) prospectively assessed reproducibility of the 169 standardized features against a public dataset of CT, PET, and MR images from 51 sarcoma patients. More than 97% of the features studied reached an excellent reproducibility (ICC > 0.9), showing the value of feature standardization in reducing variability between different feature extraction software.

The study identified several causes of deviation. For instance, lesion volumes can be represented by simple voxel cubes or polygonal models (or meshes). This affects the computation of surface area and thus morphological features. Sometimes, there are “holes”, which are dark regions inside segmented lesions. The decision whether to fill such small holes prior to feature computation can influence the computed value. Differences of this kind are controllable and can be reduced or eliminated through feature standardization.

### Feature Distribution Harmonization – Combat

Image acquisition-induced variations in radiomics features are intensively discussed in the early section of Image acquisition, where the suggested solutions to reduce such variability are mainly focused on obtaining consistent and/or comparable images through controlling image acquisition protocols and/or post-processing of acquired images using both conventional and AI-based methods.

Recently, a new data-driven method based on the empirical Bayes frameworks, called ComBat harmonization, was introduced into radiomics to reduce feature variability caused by scanners and scanning parameters (107–109). This method was initially developed for large-scale genomic data analysis (110). When combining different datasets collected from microarray experiments, a big challenge is to remove non-biological variations caused by the systematic technical differences while handling samples, i.e., to remove the so-called “batch effects”, where the batches denote operators, array types, etc. In radiomics, batches refer to scanners, imaging protocols, individual imaging parameters, etc. Unlike the imaging harmonization, the ComBat method operates directly on the computed feature values to remove batch-induced bias. This eliminates/reduces, for example, the demands for sharing and transferring medical images between institutions that can be limited by specific regulations and standardizing image acquisition settings that can be hard to be implemented in routine clinical practice.

**Figure 7:**
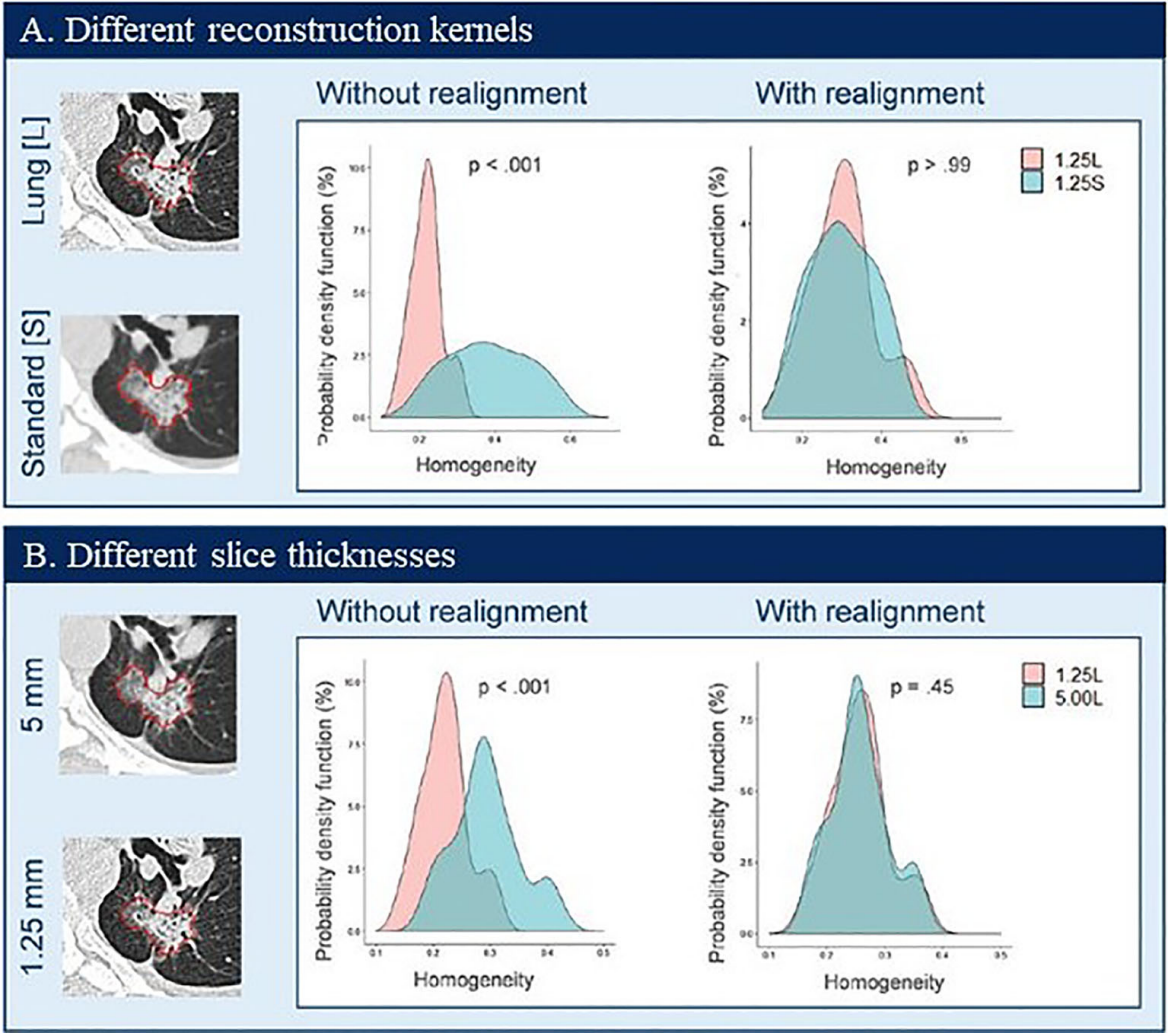
Probability density distributions of Homogeneity before (without realignment) and after (with realignment) ComBat in patient data by using two CT reconstruction kernels **(A**) and two slice thicknesses **(B)**. Reproduced with permission from (108).

**Figure 7** shows two examples of harmonization/realignment of a feature, GLCM Homogeneity (108). The example shows two distributions of the feature, computed from the images reconstructed at two different reconstruction kernels, Lung vs. Standard (example 1, **Figure 7A**), and at two different slice thicknesses, 1.25 mm vs. 5 mm (example 2, **Figure 7B**). In each example, feature distributions were better overlapped after applying the ComBat harmonization function (https://github.com/Jfortin1/ComBatHarmonization). A follow-up study independently verified the published results by applying the ComBat method to harmonize a larger set of radiomics features computed from a broader range of imaging protocols in a larger cohort of patients. The investigators noticed that the harmonization also increased the repeatability of texture features (109). This promising technology warrants validation for its clinical usefulness in radiomics.

### Experience With Open Source Software and Open Source Databases in Building Radiomics Prediction Models

There are a number of free open source software packages to compute radiomics features. Based on the literature searching results, open source and in-house feature software were used almost equally frequently in the lung cancer radiomics studies published from 2014 to July 2020 (**Supplemental Materials**; excluding ~9% “not specified” software). Pyradiomics (~15%) (66) and Imaging Biomarker Explorer (IBEX) (~8%) (62) are the two most popular open source software to study radiomics.

Recently, a new radiomic feature calculator, called RaCaT, became available (111). It calculates a large number of features that are in compliance with the IBSI standard. Although the calculator can be downloaded and used without requiring any programming skills, it does not provide any Graphical User Interface. Users need to call the calculator either from their own programming environments or from the command line.

A research group recently reported its first-hand experience in building a radiomics model to predict EGFR mutation status in NSCLC patients using two open source databases, TCIA (The Cancer Imaging Archive) (112) and TCGA (The Cancer Genome Atlas) (113), and three feature extraction software packages, the open source Pyradiomics (1319 features) and IBEX (1563 features), and an in-house package (1160 features) (114). Although they encountered some obstacles, they reported a smooth experience overall with the public datasets and open source feature extraction software. They were able to collect both image data and clinical data for the majority of patients satisfying the inclusion criteria of their study. However, the TCGA-LUAD and the TCGA-LUSC datasets contained image data and genomic data that were stored separately on the TCIA and the TCGA, respectively, for the majority of cases. In addition, the genomic data was often incomplete, which reduced the number of useable cases. The two open-source software packages had clear instructions that made them amenable to beginners. Radiomics feature definitions were well documented and were able to be extracted from the majority of lesions. Some errors did occur during the extraction in both open-source software packages that could not solved. The study found that although the three software packages selected different features to build their prediction models, the models’ performances were similar. The correlations found between those selected features by the different software indicate that these features may describe similar tumor imaging phenotypes that are associated with underlying biological characteristics.

### Discussion: Potential Strategies and Practical Considerations to Improve Feature Extraction

Variations in feature computation are caused by possible differences in feature definition, parameter setting, and implementation. Variations also come from the previous steps of image acquisition, lesion segmentation, and image preprocessing, which exaggerate variability in radiomics features and models built using these features (12, 115, 116).

*Feature Definition Standardization*. One way to reduce feature variability, enhance collaboration, and accelerate the development and validation of radiomics signatures is to standardize feature definition, parameter setting, and implementation. The IBSI’s effort in standardizing the feature extraction process is a significant step toward increasing feature transparency, reducing feature variation, and providing reference images and reference feature values to help verify/calibrate feature extraction software developed by researchers globally (104). Customizable 3D DROs can be created to help standardize radiomics features and uncover coding errors (105). It should be noted that promoting feature standardization does not mean that investigators shouldn’t develop and use their own feature definitions, parameter settings, and implementation methods that are different than those suggested by the IBSI.

*Feature Parameter Setting*. Normally, we only use about 100 or less fundamental radiomics features. However, with multiple settings of feature-specific parameters, different implementation methods, and various image pre-processing methods, the total number of features that can be provided by a feature extraction software package can easily reach multiple thousands. Currently, the settings of many feature parameters are “randomly” chosen or simply adopted from the literature where the image types and contents can be very different than those of the investigators’ own clinical studies. As a result, the same features, same feature parameter settings, and/or same image pre-processing methods are often used to study different clinical questions for different disease sites using different imaging modalities. This so called one-size-fits-all scenario may delay or prevent the discovery of radiomics signatures. In order to increase the opportunity to identify biologically relevant features while studying lung cancer, for instance, understanding the density range of lung tumors and image noise characteristics may help choose proper values of feature parameters.

*Feature Redundancy*. On one hand, multiple parameter values allow quantification of lesion textures at different scales, contrasts, and directions, which can increase the chance to identify biologically relevant features. On the other hand, multiple parameters can drastically increase the total number of features, many of which are correlated. The high dimensionality of features can also lead to model overfitting. Reducing feature dimension is necessary prior to building prediction models using machine learning methods. Feature reduction and identification of potential confounding variables such as image acquisition parameters (e.g., slice thickness) and clinically used prognosticators (e.g., tumor size) are beyond the discussions of this review paper.

*Feature Transparency*. Inadequate descriptions of feature extraction in the current literature is a big burden for widespread adoption of the features and replication/validation of the developed radiomics signatures. For researchers who are capable of writing their own feature extraction algorithms, it is important for them to track their changes of the codes using version control software and describe the feature extraction details as much as possible in publications. For the groups offering open source feature extraction software, the software version numbers along with the release dates and upgrades should be clearly documented and provided for the purposes of record tracking.

*Image Pre-Processing*. Image pre-processing includes, but is not limited to, smoothing, sharpening, and/or resampling of images prior to feature extraction. Generally speaking, image smoothing can improve density-based feature reproducibility. For instance, the LoG (Laplacian of Gaussian) texture features computed from the same-day repeat CT scan images reconstructed at different imaging settings is an example (**Figure 4A**). LoG_s1 denotes no pre-processing and LoG_s4 indicates that a large Gaussian kernel is applied to strongly smooth the original images before the feature calculation. The reproducibility of LoG features calculated on the smoothed images is drastically improved (CCC heat map colors changed from dark to bright) even when the features are calculated from images reconstructed using different kernels. However, over smoothing can suppress image texture details, which may lose clinically useful information related to low contrast textures. There is a trade-off between reproducible features and informative features.

Another image pre-processing operation is to resample CT images to isotropic resolutions in x-, y-, and z-directions. Studies show that isotropic resolutions can improve feature reproducibility (42). It is worth mentioning that, in 3D image segmentation, the isotropic resampling of images is often a precondition for direct use of 3D image processing operators that are employed by many 3D segmentation algorithms.

*Reproducible and Reliable Features*. Both re-imaging and re-segmentation can introduce variation into radiomics features. To assess the reproducibility due to re-imaging, features are extracted from a set of lesions imaged and segmented from two repeat scans acquired within a short time interval. To assess reproducibility due to re-segmentation, features need to be extracted from a set of lesions segmented by the same radiologist in at least two different sessions (intra-reader variability) and/or by at least two independent radiologists (inter-reader variability). If repeat scan image data are available, re-segmentation of lesions on repeat images can take into the account the variability caused by both re-imaging and re-segmentation simultaneously. The concordance correlation coefficient (CCCs) is a widely accepted statistical method to assess the reproducibility of radiomics features (117). Only reproducible features will be retained for the subsequent machine learning analysis. Once features are extracted, checking outliers for each feature is a practical way to help identify imaging artifacts, segmentation errors, etc.

*ComBat Feature Harmonization*. The ComBat is an easy-to-use and fast feature harmonization method recently introduced to remove batch effects in radiomics. Based on calculated feature values, the ComBat method has the ability to adjust for the batch effects at multiple layers, e.g., at institution, scanner, imaging protocol and individual imaging parameter levels. With the ComBat method, more features can become robust and be analyzed, historical image data can be better reanalyzed and multi-center data can be properly combined and/or compared. Future research includes, for instance, incorporating clinical and biological variables into the ComBat method to preserve biological variation while maximally removing batch effects. The ComBat feature harmonization opens a new and efficient avenue to accelerate the development, validation and dissemination of robust and generalized radiomics signatures and their transfers to clinical practice.

*CNN Features*. Given sufficient data, features derived from a CNN can be expected to overcome the limitations of pre-defined traditional radiomics features because a CNN’s backward propagation of errors for training purposes enables the network to self-learn novel features which are most useful for a specific application. The automated learning and iterative image filtering performed by a CNN may also make the CNN models less likely to be confounded by heterogeneous image acquisition settings. The CNN also eliminates the step of lesion segmentation, a major source of variation in radiomics. Nevertheless, radiomics can build tumor imaging phenotype models using small datasets, a necessity for many medical studies. Radiomics signatures can often be intuitively interpreted, which also makes radiomics favorable over the “black box” approach of using a CNN. In the foreseeable future, there is no doubt that both radiomics and AI/CNN will be mainstream approaches to study quantitative imaging biomarkers in precision medicine.

## Summary

Radiomics has shown promise for a variety of clinical applications in lung and other cancers, and in particular for diagnosis, prognosis, and response assessment. Radiomics derives strength from hypothesis neutral techniques that can identify subtle details or changes in patterns/features of medical images that are associated with biological activities and clinical outcomes. This, however, also creates a potential weakness: the values of computed radiomics features and the performance of radiomics models incorporating them can be sensitive to many variables intrinsic to the radiomics workflow. Given heterogeneous image acquisition settings, varied quantification software packages, different diseases’ characteristics, and small and mixed patient populations, the development of reproducible and generalizable radiomics signatures is not as straightforward as it initially appeared. Indeed, radiomics is a multidisciplinary research field. Its success relies on close collaborations among physicians, medical imaging physicists, biomedical engineers, statisticians, and computer scientists. Over the past years, great community efforts have been made to better understand sources of variation, improving reproducibility and reliability of radiomics features and models through imaging and feature harmonization and increasing transparency and quality of radiomics studies. Ever-growing open source imaging and genomic databases as well as open source software packages help accelerate the development and external validation of radiomics signatures.

## Author Contributions

The author confirms being the sole contributor of this work and has approved it for publication.

## Funding

The author would like to acknowledge the support from National Institute of Health (Award Number U01 CA225431). 

## Conflict of Interest

The author BZ receives royalties from Varian Medical Systems and Keosys Medical Imaging and funding from NIH.
