# Supplementary material for: Understanding Sources of Variation to Improve the Reproducibility of Radiomics
Source: Front Oncol. 2021 Mar 29;11:633176. doi: 10.3389/fonc.2021.633176 (PMC8039446; doi:10.3389/fonc.2021.633176)
Supplement: Supplementary file 1 [file DataSheet_1.pdf]

Supplementary Materials

Literature search

Search strategy

Our aim was to extend the search performed by Fornacon-Wood et al. [1]. We used similar criteria in the paper and identified publications since January 2020 that report radiomics analyses on non-small cell lung cancer (NSCLC) patients with the aim of predicting patient outcome using PubMed database. We used a combination of the following key words a) "radiomic" or "radiomics" and "lung cancer" and b) "cancer" and c) "standardization" or "reliable" or "impact of" or "improvement" or "repeatable" or "reproducible" or "repeatability" or "reproducibility" or "test–retest" or "variability" or "limitation" or "limitations" or "vulnerability" or "vulnerabilities" or "stability" or "stable" or "robustness" or "robust" or "quality" or "agreement" or "effect of". The search was done on July 27th, 2020 and start date was set to January 1st, 2020

Search Outcomes

The search resulted in 38 publications and 1 study not found from the search was included into the results. We then screened the titles and abstracts to identify unique studies that aimed to create predictive radiomics models for clinical use in NSCLC patients from CT images. Inclusion criteria were publications assessing outcomes of overall survival, metastases, treatment-induced toxicities or finding biological correlations. Studies using a modality other than CT, where the primary cancer was not NSCLC and review articles were not included in this step. Exclusion criteria included CT studies not from planning CT, CBCT or diagnostic CT, if access to the article could not be gained, if the article was in a language other than English, if the study included deep learning as opposed to the traditional radiomics workflow discussed in this review, and studies predicting nodule malignancy. Studies of analysis reproducibility or methodology limitations were also excluded from this search. After applying the inclusion and the exclusion criteria, there were 15 new papers to add to the previous collection of data. See Fornacon-Wood's supplementary information for more information on the previously collected data.

In our Supplementary Table, radiomics studies in NSCLC split into sections with an addition of papers published in 2020 at the end. The Table is an extension to a combined form of Supplementary Table 2 and Supplementary Table 3 from Fornacon-Wood's 2020 paper [1]. The data taken from them have been *italicized*, and we added the 15 newly identified publications from 2020 to the dataset. We then added more information to the dataset on whether the study reported on the following: slice thicknesses, reconstruction kernels, use of test-retest, use of inter/intra-reader validation tests, which segmentation software was used, if segmentations were supervised, and the number of features extracted.

We collected information about how many radiomics studies reported elements that can impact the reproducibility of the study from the total of 78 studies published between January 2014 to June 2020. The elements we looked for in each study to report were slice thickness, reconstruction kernel, use of test-retest data, testing inter/intra-reader variability, and having supervised segmentation. Once the tables were organized, we counted the number of studies that reported each element in their study for each year from 2014 to 2020, with the year 2020 only including studies that were published from January to June. We also recorded the total number of studies that mentioned each category for all the years from 2014 to 2020 and calculated the percentages of those studies out of 78 total.

We then separated the studies into four groups for different combinations of reporting slice thickness and/or image reconstruction kernel. For example, a study was assigned to Group 1 if it reported both slice thickness and image reconstruction kernel while a study that reported neither elements was assigned to Group 2. A study that reported only slice thickness was assigned to Group 3, and a study that reported only reconstruction kernel was assigned to Group 4. Similarly, studies were separated into 4 groups based on their reporting of using test-retest data and/or testing inter/intra-reader variability.

The graphs in the review article (Fig. 2) were created as combination graphs to display both the total count per year and the percentage per year. The x-axis was labeled with years, left y-axis was labeled with the number of studies, and the right y-axis was labeled with the percentage of studies. The total number of studies for each year is included in every graph with a dark blue bar for reference. For each element, there were two graphs of the same color: a bar graph displaying the number of studies that reported that element and a line graph displaying the percentage of studies for that year that reported that element. The value of each point/bar is labeled next to its respective graph.

Supplementary Table 1. Radiomics studies in NSCLC

Table 1 is an extension to a combined form of Supplementary Table 2 and Supplementary Table 3 from Fornacon-Wood's 2020 paper [1].

Reference	***NSCLC stage***	***Endpoint (for clinical)***	**Slice thickness**	**Reconstruction kernel**	***Test-retest dataset used***	**Inter- and intra-reader variability**	**Segmentation software**	**Supervised Segmentation**	***Feature extraction software***
Aerts et al. 2014 [2]	*1−3b*	*OS*	Lung1: 3mm, Lung2: not specified, Lung3: 1.5-5mm, H&N1: 3mm, H&N2: 2.5mm	not specified	*RIDER*	inter	manual	yes	*In-house*
Van Timmeren et al. 2017 [3]	*1−4*	*OS*	Dataset 1: 3mm, Dataset 2: 3mm, Dataset 3: 2.5mm - 3mm	not specified	*not specified*	not specified	manual	yes	*In-house*

Grossman et al. 2017 [4]	*1−3*	*OS*	not specified	not specified	*not specified*	not specified	not specified	not specified	*Pyradiomics and in-house*
Yu et al. 2017 [5]	*1*	*OS*	2.5mm	standard reconstruction kernel	*not specified*	inter	3D slicer manual	yes	*IBEX*
Chaddad et al. 2017 [6]	*1−3b*	*OS*	Lung1: 3mm	not specified	*not specified*	not specified	manual	yes	*In-house*
Fave et al. 2017 [7]	*3*	*OS*	2.5mm	not specified	*not specified*	not specified	in-house	yes	*IBEX*
Li et al. 2017 [8]	*1−2a*	*OS*	2.5mm or 3mm	not specified	*not specified*	intra	in-house	yes	*Definiens developer*
Li et al. 2017 [9]	*1−2a*	*OS*	3mm	not specified	*not specified*	intra	in-house	yes	*Definiens developer*
Tang et al. 2018 [10]	*1−3*	*OS*	not specified	not specified	*not specified*	inter	3D slicer, MIM manual, manual	yes	*IBEX*
Bianconi et al. 2018 [11]	*1−3*	*OS*	not specified	not specified	*not specified*	not specified	manual	yes	*not specified*
De Jong et al. 2018 [12]	*4*	*OS*	3mm	not specified	*not specified*	not specified	Eclipse manual	yes	*in-house and CERR*
Lee et al. 2018 [13]	*1−3*	*OS*	1.25mm or 0.625mm	not specified	*not specified*	inter	in-house	yes	*in-house*
He et al. 2018 [14]	*1−3*	*OS*	0.625mm - 3mm	not specified	*not specified*	not specified	In-house	yes	*Pyradiomics*
Starkov et al. 2018 [15]	*1*	*OS*	1.25mm or 2.5mm	not specified	*not specified*	not specified	manual	yes	*in-house*
Yang et al. 2018 [16]	*1−4*	*OS*	training: 3mm, external validation: 1mm-5mm	not specified	*not specified*	both	3D Slicer manual	yes	*in-house*
Wang et al. 2019 [17]	*3*	*OS*	0.625mm	not specified	*not specified*	intra	in-house	yes	*pyradiomics*
Shi et al. 2019 [18]	*3*	*OS*	3mm, 1mm	not specified	*in-house*	not specified	RayStation	yes	*IBEX*
Van Timmeren et al. 2019 [19]	*1−4*	*OS*	Dataset 1: 3mm, Dataset 2: 3mm, Dataset 3: 2mm or 5mm, Dataset 4: 2.5mm or 3mm	All 4 datasets reconstrcuted. See protocol.	*not specified*	not specified	CBCT: REGGUI, Dataset 3 and 4: MIM manual	yes	*in-house*
Huang et al. 2019 [20]	*1−4*	*OS*	not specified	B31f or B30f	*RIDER*	not specified	MIM manual, 3D Slicer manual	yes	*in-house*
Franceschini et al. 2019 [21]	*1−2*	*OS*	not specified	not specified	*not specified*	not specified	Eclipse manual	yes	*LIFEx*
Coroller et al. 2015 [22]	*2−3*	*tumor recurrence*	most commonly 2.5mm	not specified	*not specified*	not specified	eclipse manual	yes	*In-house and CERR*
Mattonen et al. 2016 [23]	*1*	*tumor recurrence*	pre-treatment: 2.5mm, post-treatment: 2.5mm - 5mm	not specified	*not specified*	inter	in-house	not specified	*In-house*
Huynh et al. 2016 [24]	*1−2*	*tumor recurrence, OS*	2.5mm	not specified	*RIDER*	not specified	Eclipse manual	yes	*In-house and 3D Slicer*
Huynh et al. 2017 [25]	*1−2a*	*tumor recurrence*	2.5mm	AIP (average intensity projection) and FB (free breathing) standard reconstruction kernel	*RIDER*	not specified	Eclipse manual	yes	*In-house and 3D Slicer*

Dou et al. 2018 [26]	*2−3*	*tumor recurrence*	2.5mm - 5mm	standard reconstruction kernel	*RIDER*	not specified	Eclipse manual	yes	*Pyradiomics*
Ferreira Junior et al. 2018 [27]	*1−4*	*tumor recurrence*	1mm - 1.5mm	not specified	*not specified*	not specified	3D slicer Growcut	not specified	*IBEX*
Yang et al. 2018 [28]	*1−3*	*tumor recurrence*	2mm	not specified	*not specified*	not specified	3D Slicer U-net	not specified	*Pyradiomics*
Zhong et al. 2018 [29]	*1−2*	*tumor recurrence*	1mm	not specified	*not specified*	not specified	in-house, manual	yes	*MaZda*
Lafata et al. 2019 [30]	*1*	*tumor recurrence*	2mm or 2.5mm	FBP reconstruction	*not specified*	not specified	manual	yes	*In-house*
Akinci D'Antonoli et al. 2019 [31]	*1−2b*	*tumor recurrence*	2.5mm	standard kernel	*not specified*	not specified	in-house	yes	*Moddicom*
He et al. 2019 [32]	*Not specified*	*tumor recurrence*	1.25mm	not specified	*not specified*	inter	manual	yes	*In-house*
Xu et al. 2019 [33]	*3−4*	*tumor recurrence*	1mm	not specified	*RIDER*	not specified	MIM manual	yes	*in-house*
Cong et al. 2019 [34]	*1a*	*tumor recurrence*	1mm	not specified	*not specified*	both	ITK-SNAP	yes	*Artificial intelligence kit*
Coroller et al. 2016 [35]	*2−3*	*DFS, PFS*	average 3mm	not specified	*RIDER*	not specified	Eclipse manual	yes	*in-house*
Huang et al. 2016 [36]	*1−2*	*DFS, PFS*	2.5mm	not specified	*not specified*	inter	in-house	not specified	*in-house*
Song et al. 2016 [37]	*1−4*	*DFS, PFS*	2.5mm	not specified	*not specified*	not specified	TBGA	yes	*in-house*
Coroller et al. 2017 [38]	*2−3*	*DFS, PFS*	3mm	not specified	*RIDER*	not specified	Eclipse manual	yes	*in-house*
Tunali et al. 2019 [39]	*3b-4*	*DFS, PFS*	3mm	B41f	*RIDER*	not specified	Definiens	yes	*in-house*
Moran et al. 2017 [40]	*1*	*Lung Injury*	1mm, 1.25mm, 1.5mm	lung B60 or I70	*not specified*	not specified	Pinnacle manual	yes	*in-house*
Krafft et al. 2018 [41]	*Not specified*	*Lung Injury*	2.5mm	standard convolution kernel	*not specified*	not specified	not specified	not specified	*in-house*
Yuan et al. 2018 [42]	*1*	*stage 1*	1mm	B60	*not specified*	not specified	lungCAD	yes	*Artificial intelligence kit*
Yang et al. 2019 [43]	*1−3*	*dry pleural dissemination*	1mm	B31f	*not specified*	not specified	3D slicer Growcut	yes	*Pyradiomics*
Aerts et al. 2016 [44]	*Early stage*	*EGFR*	1.25mm	lung kernel	*RIDER*	not specified	in-house	not specified	*Pyradiomics*
Rios Velazquez et al. 2017 [45]	*1−4*	*EGFR, KRAS*	range 1-7mm	not specified	*RIDER*	not specified	3D slicer, in-house	not specified	*in-house*
Mei et al. 2018 [46]	*Not specified*	*EGFR*	2mm with 1mm increment, 1.5mm with 0.75mm increment	not specified	*not specified*	not specified	not specified	yes	*pyradiomics*
Digumarthy et al. 2019 [47]	*Not specified*	*EGFR*	2.5mm, 3mm	standard soft tissue reconstruction	*not specified*	not specified	in-house, manual	yes	*CT texture analysis*
Jia et al. 2019 [48]	*1−4*	*EGFR*	1mm, 5mm	not specified	*not specified*	not specified	TPS Pinnacle	yes	*not specified*
Li et al. 2019 [49]	*1−4*	*EGFR subtypes (19Del and L858R)*	1mm, 2mm	B convolution kernel	*not specified*	inter	ITK-SNAP, manual	yes	*in-house*
Tu et al. 2019 [50]	*1−4*	*EGFR*	1mm	high and standard resolution	*not specified*	both	ITK-SNAP, manual	yes	*in-house*
Yang et al. 2019 [51]	*Not specified*	*EGFR*	2mm	B30 or I30	*not specified*	not specified	3D Slicer U-net	yes	*Pyradiomics*
Wang et al. 2019 [52]	*1−2*	*EGFR, TP53*	1.25mm	standard	*not specified*	not specified	`	yes	*Pyradiomics, IBSI*

Bak et al. 2018 [53]	*1−4*	*Various*	2.5mm - 5mm	standard soft tissue reconstruction	*not specified*	not specified	MRIcro manual	yes	*in-house*
Patil et al. 2016 [54]	*Not specified*	*ADC, LCC, SCC, NOS*	not specified	not specified	*not specified*	not specified	not specified	not specified	*in-house*
Wu et al. 2016 [55]	*1−4*	*ADC, SCC*	not specified	not specified	*not specified*	not specified	manual	not specified	*in-house*
Zhu et al. 2018 [56]	*Not specified*	*ADC, SCC*	not specified	not specified	*not specified*	inter	ITK-SNAP manual	yes	*in-house*
E et al. 2019 [57]	*Not specified*	*ADC, SCC, SCLC*	CECT: 1mm, noncontrast CT: 1-5mm	B30f	*RIDER*	not specified	Weasis, in-house	yes	*in-house*
Liu et al. 2019 [58]	*Not specified*	*ADC, LCC, SCC, NOS*	1.5-5mm	not specified	*not specified*	not specified	manual	yes	*not specified*
Zhou et al. 2018 [59]	*1−4*	*Ki-67*	1.25mm	not specified	*not specified*	not specified	manual	yes	*3D slicer*
Gu et al. 2019 [60]	*Not specified*	*Ki-67*	5mm	not specified	*not specified*	not specified	manual MaZda	yes	*MaZda*
Song et al. 2017 [61]	*1−3*	*Micropapillary pattern*	2.5-3.5mm	not specified	*not specified*	inter	in-house	yes	*not specified*
Chen et al. 2018 [62]	*Not specified*	*Degree of differentiation*	1.25mm	not specified	*not specified*	not specified	ImageJ manual	yes	*in-house*
She et al. 2018 [63]	*Not specified*	*Invasive vs non-invasive adenocarcinoma*	1mm	Siemens: standard B31 filter, Philips: sharp C filter	*not specified*	not specified	manual	yes	*in-house*
Yang et al. 2019 [64]	*Not specified*	*Invasive vs non-invasive adenocarcinoma*	5mm	not specified	*not specified*	both	manual ITK-SNAP	yes	*Artificial intelligence kit*
Lin et al. 2020 [65]	0 - 2a	OS, DFS	0.625 - 2mm	Siemens: B20f/B30f/B31f/Br44d, GE: standard, Philips: B/C, Toshiba: F03/F04	RIDER	inter	in-house	yes	In-house
Dissaux et al. 2020 [66]	early stage	tumor recurrence	0.5mm - 3.27mm	not specified	not specified	not specified	MIM manual	yes	not specified
Mu et al. 2020 [67]	3b -4	PFS, OS	<5 mm	not specified	not specified	not specified	manual	yes	not specified
Nardone et al. 2020 [68]	3b -4	OS, PFS	2.5mm	standard	not specified	both	manual	yes	Lifex
Wang et al. 2020 [69]	1--4	survival	0.8mm - 3mm	not specified	not specified	not specified	in-house	yes	not specified
Qin et al. 2020 [70]	1	PFS	2.5mm	not specified	not specified	not specified	manual	yes	IBEX
Li et al. 2020 [71]	4	PFS	1mm	not specified	not specified	not specified	manual ITK-SNAP	yes	in-house
Khorrami et al. 2020 [72]	Ia - 2a	tumor recurrence	0.6 mm - 5mm	Siemens: ‘B321f’, ‘B35f’; Philips: ‘B’, ‘C’, ‘D’; Toshiba: ‘FC01’, ‘FC08’, ‘FC18’; GE: ‘SOFT’, ‘STANDARD’, Siemens: ‘B321s’, ‘B35fs; GE: ‘LUNG’	RIDER	not specified	3D Slicer manual	yes	not specified
Farchione et al. 2020 [73]	1a - 3	OS	1.25mm, 2.5mm	standard and bone	not specified	not specified	manual Eclipse	yes	moddicom

Kadoya et al. 2020 [74]	1 - 3b	OS	1.25mm - 3mm	not specified	RIDER	not specified	3D slicer	yes	pyradiomics
Dercle et al. 2020 [75]	1--4	EGFR	<10mm	lung	not specified	not specified	in-house	yes	In-house
Mazzaschi et al. 2020 [76]	1-3a	OS, DFS	1mm - 2.5mm	not specified	not specified	inter	3D Slicer manual	yes	3D slicer
Vuong et al. 2020 [77]	3a, 3b	OS	2mm, 3.27mm	GE – STANDARD, Siemens – B30f/B31f, Toshiba – FC18, and Philips – B, validation: FBP and a smooth kernel (STANDARD, I30f, B31f)	not specified	inter	MIM manual	yes	in-house
Botta et al. 2020 [78]	1-3a	OS	2.5mm	Body, standard, filtered back projection, iterative reconstruction	in-house	not specified	manual	yes	IBEX
Kim et al. 2020 [79]	1 -- 4	DFS	1mm - 5mm	not specified	not specified	inter	in-house	yes	Pyradiomics and in-house

References

1. Fornacon-Wood I, Faivre-Finn C, O'Connor JP, Price GJ. Radiomics as a personalized medicine tool in lung cancer: Separating the hope from the hype. Lung Cancer. 2020;146:197-208. doi: 10.1016/j.lungcan.2020.05.028
2. Aerts HJWL, Velazquez ER, Leijenaar RTH, et al. Decoding tumour phenotype by noninvasive imaging using a quantitative radiomics approach. Nat Commun. 2014;5(4006):4006. doi:10.1038/ncomms5006
3. van Timmeren JE, Leijenaar RTH, van Elmpt W, et al. Survival prediction of non-small cell lung cancer patients using radiomics analyses of cone-beam CT images. Radiother Oncol. 2017;123(3):363-369. doi:10.1016/j.radonc.2017.04.016
4. Grossmann P, Stringfield O, El-Hachem N, et al. Defining the biological basis of radiomic phenotypes in lung cancer. Elife. 2017;6:1-22. doi:10.7554/eLife.23421
5. Yu W, Tang C, Hobbs BP, et al. Development and Validation of a Predictive Radiomics Model for Clinical Outcomes in Stage I Non-small Cell Lung Cancer. Int J Radiat Oncol Biol Phys. 2017;102(4):1090-1097. doi:10.1016/j.ijrobp.2017.10.046
6. Chaddad A, Desrosiers C, Toews M, Abdulkarim B. Predicting survival time of lung cancer patients using radiomic analysis. Oncotarget. 2017;8(61):104393-104407. doi:10.18632/oncotarget.22251
7. Fave X, Zhang L, Yang J, et al. Delta-radiomics features for the prediction of patient outcomes in non-small cell lung cancer. Sci Rep. 2017;7(1):588. doi:10.1038/s41598-017-00665-z

8. Li Q, Kim J, Balagurunathan Y, et al. CT imaging features associated with recurrence in non-small cell lung cancer patients after stereotactic body radiotherapy. *Radiat Oncol*. 2017;12(1):158. doi:10.1186/s13014-017-0892-y
9. Li Q, Kim J, Balagurunathan Y, et al. Imaging features from pretreatment CT scans are associated with clinical outcomes in nonsmall-cell lung cancer patients treated with stereotactic body radiotherapy. *Med Phys*. 2017;44(8):4341-4349. doi:10.1002/mp.12309
10. Tang C, Hobbs B, Amer A, et al. Development of an Immune-Pathology Informed Radiomics Model for Non-Small Cell Lung Cancer. Sci Rep. 2018;8(1):1922. doi:10.1038/s41598-018-20471-5
11. Bianconi F, Fravolini ML, Bello-Cerezo R, Minestrini M, Scialpi M, Palumbo B. Evaluation of shape and textural features from CT as prognostic biomarkers in non-small cell lung cancer. *Anticancer Res*. 2018;38(4):2155-2160. doi:10.21873/anticanres.12456
12. de Jong EEC, van Elmpt W, Rizzo S, et al. Applicability of a prognostic CT-based radiomic signature model trained on stage I-III non-small cell lung cancer in stage IV non-small cell lung cancer. *Lung Cancer*. 2018;124:6-11. doi:10.1016/j.lungcan.2018.07.023
13. Lee G, Park H, Sohn I, et al. Comprehensive Computed Tomography Radiomics Analysis of Lung Adenocarcinoma for Prognostication. *Oncologist*. 2018;23(7):806-813. doi:10.1634/theoncologist.2017-0538
14. He B, Zhao W, Pi JY, et al. A biomarker basing on radiomics for the prediction of overall survival in non-small cell lung cancer patients. *Respir Res*. 2018;19(1):199. doi:10.1186/s12931-018-0887-8
15. Starkov P, Aguilera TA, Golden DI, et al. The use of texture-based radiomics CT analysis to predict outcomes in early-stage non-small cell lung cancer treated with stereotactic ablative radiotherapy. *Br J Radiol*. 2019;92(1094):20180228. doi:10.1259/bjr.20180228
16. Yang L, Yang J, Zhou X, et al. Development of a radiomics nomogram based on the 2D and 3D CT features to predict the survival of non-small cell lung cancer patients. *Eur Radiol*. 2019;29(5):2196-2206. doi:10.1007/s00330-018-5770-y
17. Wang L, Dong T, Xin B, et al. Integrative nomogram of CT imaging, clinical, and hematological features for survival prediction of patients with locally advanced non-small cell lung cancer. *Eur Radiol*. 2019;29(6):2958-2967. doi:10.1007/s00330-018-5949-2
18. Shi L, Rong Y, Daly M, et al. Cone-beam computed tomography-based delta-radiomics for early response assessment in radiotherapy for locally advanced lung cancer. *Phys Med Biol*. 2019;65(1):15009. doi:10.1088/1361-6560/ab3247
19. van Timmeren JE, van Elmpt W, Leijenaar RTH, et al. Longitudinal radiomics of cone-beam CT images from non-small cell lung cancer patients: Evaluation of the added prognostic value for overall survival and locoregional recurrence. *Radiother Oncol*. 2019;136:78-85. doi:10.1016/j.radonc.2019.03.032
20. Huang L, Chen J, Hu W, et al. Assessment of a Radiomic Signature Developed in a General NSCLC Cohort for Predicting Overall Survival of ALK-Positive Patients With Different Treatment Types. *Clin Lung Cancer*. 2019;20(6):e638-e651. doi:10.1016/j.cllc.2019.05.005
21. Franceschini D, Cozzi L, De Rose F, et al. A radiomic approach to predicting nodal relapse and disease-specific survival in patients treated with stereotactic body radiation therapy for

early-stage non-small cell lung cancer. *Strahlentherapie und Onkol*. November 2019. doi:10.1007/s00066-019-01542-6
22. Coroller TP, Grossmann P, Hou Y, et al. CT-based radiomic signature predicts distant metastasis in lung adenocarcinoma. *Radiother Oncol*. 2015;114(3):345-350. doi:10.1016/j.radonc.2015.02.015
23. Mattonen SA, Palma DA, Johnson C, et al. Detection of Local Cancer Recurrence after Stereotactic Ablative Radiation Therapy for Lung Cancer: Physician Performance Versus Radiomic Assessment. *Int J Radiat Oncol Biol Phys*. 2016;94(5):1121-1128. doi:10.1016/j.ijrobp.2015.12.369
24. Huynh E, Coroller TP, Narayan V, et al. CT-based radiomic analysis of stereotactic body radiation therapy patients with lung cancer. *Radiother Oncol*. 2016;120(2):258-266. doi:10.1016/j.radonc.2016.05.024
25. Huynh E, Coroller TP, Narayan V, et al. Associations of radiomic data extracted from static and respiratory-gated CT scans with disease recurrence in lung cancer patients treated with SBRT. *PLoS One*. 2017;12(1):e0169172. doi:10.1371/journal.pone.0169172
26. Dou TH, Coroller TP, van Griethuysen JJM, Mak RH, Aerts HJWL. Peritumoral radiomics features predict distant metastasis in locally advanced NSCLC. *PLoS One*. 2018;13(11):e0206108. doi:10.1371/journal.pone.0206108
27. Ferreira Junior JR, Koenigkam-Santos M, Cipriano FEG, Fabro AT, Azevedo-Marques PM de. Radiomics-based features for pattern recognition of lung cancer histopathology and metastases. *Comput Methods Programs Biomed*. 2018;159:23-30. doi:10.1016/j.cmpb.2018.02.015
28. Yang X, Pan X, Liu H, et al. A new approach to predict lymph node metastasis in solid lung adenocarcinoma: A radiomics nomogram. *J Thorac Dis*. 2018;10(Suppl 7):S807-S819. doi:10.21037/jtd.2018.03.126
29. Zhong Y, Yuan M, Zhang T, Zhang YD, Li H, Yu TF. Radiomics approach to prediction of occult mediastinal lymph node metastasis of lung adenocarcinoma. *Am J Roentgenol*. 2018;211(1):109-113. doi:10.2214/AJR.17.19074
30. Lafata KJ, Hong JC, Geng R, et al. Association of pre-treatment radiomic features with lung cancer recurrence following stereotactic body radiation therapy. *Phys Med Biol*. 2019;64(2). doi:10.1088/1361-6560/aaf5a5
31. Akinci D'Antonoli T, Farchione A, Lenkowicz J, et al. CT Radiomics Signature of Tumor and Peritumoral Lung Parenchyma to Predict Nonsmall Cell Lung Cancer Postsurgical Recurrence Risk. *Acad Radiol*. 2020;27(4):497-507. doi:10.1016/j.acra.2019.05.019
32. He L, Huang Y, Yan L, Zheng J, Liang C, Liu Z. Radiomics-based predictive risk score: A scoring system for preoperatively predicting risk of lymph node metastasis in patients with resectable non-small cell lung cancer. *Chinese J Cancer Res*. 2019;31(4):641-652. doi:10.21147/j.issn.1000-9604.2019.04.08
33. Xu X, Huang L, Chen J, et al. Application of radiomics signature captured from pretreatment thoracic CT to predict brain metastases in stage III/IV ALK-positive non-small cell lung cancer patients. *J Thorac Dis*. 2019;11(11):4516-4528. doi:10.21037/jtd.2019.11.01

34. Cong M, Feng H, Ren JL, et al. Development of a predictive radiomics model for lymph node metastases in pre-surgical CT-based stage IA non-small cell lung cancer. *Lung Cancer*. 2020;139:73-79. doi:10.1016/j.lungcan.2019.11.003
35. Coroller TP, Agrawal V, Narayan V, et al. Radiomic phenotype features predict pathological response in non-small cell lung cancer. *Radiother Oncol*. 2016;119(3):480-486. doi:10.1016/j.radonc.2016.04.004
36. Huang Y, Liu Z, He L, et al. Radiomics Signature: A Potential Biomarker for the Prediction of Disease-Free Survival in Early-Stage (I or II) Non—Small Cell Lung Cancer. *Radiology*. 2016;281(3):947-957. doi:10.1148/radiol.2016152234
37. Song J, Dong D, Huang Y, Zang Y, Liu Z, Tian J. Association between tumor heterogeneity and progression-free survival in non-small cell lung cancer patients with EGFR mutations undergoing tyrosine kinase inhibitors therapy. *Proc Annu Int Conf IEEE Eng Med Biol Soc EMBS*. 2016;2016-Octob:1268-1271. doi:10.1109/EMBC.2016.7590937
38. Coroller TP, Agrawal V, Huynh E, et al. Radiomic-Based Pathological Response Prediction from Primary Tumors and Lymph Nodes in NSCLC. *J Thorac Oncol*. 2017;12(3):467-476. doi:10.1016/j.jtho.2016.11.2226
39. Tunali I, Gray JE, Qi J, et al. Novel clinical and radiomic predictors of rapid disease progression phenotypes among lung cancer patients treated with immunotherapy: An early report. *Lung Cancer*. 2019;129:75-79. doi:10.1016/j.lungcan.2019.01.010
40. Moran A, Daly ME, Yip SSF, Yamamoto T. Radiomics-based Assessment of Radiation-induced Lung Injury After Stereotactic Body Radiotherapy. *Clin Lung Cancer*. 2017;18(6):e425-e431. doi:10.1016/j.cllc.2017.05.014
41. Krafft SP, Rao A, Stingo F, et al. The utility of quantitative CT radiomics features for improved prediction of radiation pneumonitis. *Med Phys*. 2018;45(11):5317-5324. doi:10.1002/mp.13150
42. Yuan M, Liu JY, Zhang T, Zhang YD, Li H, Yu TF. Prognostic Impact of the Findings on Thin-Section Computed Tomography in stage 1 lung adenocarcinoma with visceral pleural invasion. *Sci Rep*. 2018;8(1):4743. doi:10.1038/s41598-018-22853-1
43. Yang M, Ren Y, She Y, et al. Imaging phenotype using radiomics to predict dry pleural dissemination in non-small cell lung cancer. *Ann Transl Med*. 2019;7(12):259. doi:10.21037/atm.2019.05.20
44. Aerts HJWL, Grossmann P, Tan Y, et al. Defining a Radiomic Response Phenotype: A Pilot Study using targeted therapy in NSCLC. *Sci Rep*. 2016;6:33860. doi:10.1038/srep33860
45. Rios Velazquez E, Parmar C, Liu Y, et al. Somatic mutations drive distinct imaging phenotypes in lung cancer. *Cancer Res*. 2017;77(14):3922-3930. doi:10.1158/0008-5472.CAN-17-0122
46. Mei D, Luo Y, Wang Y, Gong J. CT texture analysis of lung adenocarcinoma: Can Radiomic features be surrogate biomarkers for EGFR mutation statuses. *Cancer Imaging*. 2018;18(1):52. doi:10.1186/s40644-018-0184-2
47. Digumarthy SR, Padole AM, Gullo R Lo, Sequist L V., Kalra MK. Can CT radiomic analysis in NSCLC predict histology and EGFR mutation status? *Medicine (Baltimore)*. 2019;98(1):e13963. doi:10.1097/MD.0000000000013963

48. Jia TY, Xiong JF, Li XY, et al. Identifying EGFR mutations in lung adenocarcinoma by noninvasive imaging using radiomics features and random forest modeling. *Eur Radiol*. 2019;29(9):4742-4750. doi:10.1007/s00330-019-06024-y
49. Li S, Ding C, Zhang H, Song J, Wu L. Radiomics for the prediction of EGFR mutation subtypes in non-small cell lung cancer. *Med Phys*. 2019;46(10):4545-4552. doi:10.1002/mp.13747
50. Tu W, Sun G, Fan L, et al. Radiomics signature: A potential and incremental predictor for EGFR mutation status in NSCLC patients, comparison with CT morphology. *Lung Cancer*. 2019;132:28-35. doi:10.1016/j.lungcan.2019.03.025
51. Yang X, Dong X, Wang J, et al. Computed Tomography-Based Radiomics Signature: A Potential Indicator of Epidermal Growth Factor Receptor Mutation in Pulmonary Adenocarcinoma Appearing as a Subsolid Nodule. *Oncologist*. 2019;24(11):e1156-e1164. doi:10.1634/theoncologist.2018-0706
52. Wang X, Kong C, Xu W, et al. Decoding tumor mutation burden and driver mutations in early stage lung adenocarcinoma using CT-based radiomics signature. *Thorac cancer*. 2019;10(10):1904-1912. doi:10.1111/1759-7714.13163
53. Bak SH, Park H, Lee HY, et al. Imaging genotyping of functional signaling pathways in lung squamous cell carcinoma using a radiomics approach. *Sci Rep*. 2018;8(1):1-9. doi:10.1038/s41598-018-21706-1
54. Patil R, Mahadevaiah G, Dekker A. An Approach Toward Automatic Classification of Tumor Histopathology of Non–Small Cell Lung Cancer Based on Radiomic Features. *Tomography*. 2016;2(4):374-377. doi:10.18383/j.tom.2016.00244
55. Wu W, Parmar C, Grossmann P, et al. Exploratory Study to Identify Radiomics Classifiers for Lung Cancer Histology. *Front Oncol*. 2016;6(71):1-11. doi:10.3389/fonc.2016.00071
56. Zhu X, Dong D, Chen Z, et al. Radiomic signature as a diagnostic factor for histologic subtype classification of non-small cell lung cancer. *Eur Radiol*. 2018;28(7):2772-2778. doi:10.1007/s00330-017-5221-1
57. E L, Lu L, Li L, Yang H, Schwartz LH, Zhao B. Radiomics for Classifying Histological Subtypes of Lung Cancer Based on Multiphasic Contrast-Enhanced Computed Tomography. *J Comput Assist Tomogr*. 2019;43(2):300-306. doi:10.1097/RCT.0000000000000836
58. Liu J, Cui J, Liu F, Yuan Y, Guo F, Zhang G. Multi-subtype classification model for non-small cell lung cancer based on radiomics: SLS model. *Med Phys*. 2019;46(7):3091-3100. doi:10.1002/mp.13551
59. Zhou B, Xu J, Tian Y, Yuan S, Li X. Correlation between radiomic features based on contrast-enhanced computed tomography images and Ki-67 proliferation index in lung cancer: A preliminary study. *Thorac Cancer*. 2018;9(10):1235-1240. doi:10.1111/1759-7714.12821
60. Gu Q, Feng Z, Liang Q, et al. Machine learning-based radiomics strategy for prediction of cell proliferation in non-small cell lung cancer. *Eur J Radiol*. 2019;118:32-37. doi:10.1016/j.ejrad.2019.06.025
61. Song SH, Park H, Lee G, et al. Imaging Phenotyping Using Radiomics to Predict Micropapillary Pattern within Lung Adenocarcinoma. *J Thorac Oncol*. 2017;12(4):624-632. doi:10.1016/j.jtho.2016.11.2230

62. Chen X, Fang M, Dong D, et al. A Radiomics Signature in Preoperative Predicting Degree of Tumor Differentiation in Patients with Non–small Cell Lung Cancer. *Acad Radiol*. 2018;25(12):1548-1555. doi:10.1016/j.acra.2018.02.019
63. She Y, Zhang L, Zhu H, et al. The predictive value of CT-based radiomics in differentiating indolent from invasive lung adenocarcinoma in patients with pulmonary nodules. *Eur Radiol*. 2018;28(12):5121-5128. doi:10.1007/s00330-018-5509-9
64. Yang B, Guo L, Lu G, Shan W, Duan L, Duan S. Radiomic signature: a non-invasive biomarker for discriminating invasive and non-invasive cases of lung adenocarcinoma. *Cancer Manag Res*. 2019;11:7825-7834. doi:10.2147/CMAR.S217887
65. Lu L, Wang D, Wang L, et al. A quantitative imaging biomarker for predicting disease-free-survival-associated histologic subgroups in lung adenocarcinoma. *Eur Radiol*. 2020;30(7):3614-3623. doi:10.1007/s00330-020-06663-6
66. Dissaux G, Visvikis D, Da-ano R, et al. Pretreatment 18F-FDG PET/CT Radiomics Predict Local Recurrence in Patients Treated with Stereotactic Body Radiotherapy for Early-Stage Non–Small Cell Lung Cancer: A Multicentric Study. *J Nucl Med*. 2020;61(6):814-820. doi:10.2967/jnumed.119.228106
67. Mu W, Tunali I, Gray JE, Qi J, Schabath MB, Gillies RJ. Radiomics of 18F-FDG PET/CT images predicts clinical benefit of advanced NSCLC patients to checkpoint blockade immunotherapy. *Eur J Nucl Med Mol Imaging*. 2020;47(5):1168-1182. doi:10.1007/s00259-019-04625-9
68. Nardone V, Tini P, Pastina P, et al. Radiomics predicts survival of patients with advanced non-small cell lung cancer undergoing PD-1 blockade using Nivolumab. *Oncol Lett*. 2020;19(2):1559-1566. doi:10.3892/ol.2019.11220Nardone et al. 2020
69. Wang X, Duan H, Li X, Ye X, Huang G, Nie S. A prognostic analysis method for non-small cell lung cancer based on the computed tomography radiomics. *Phys Med Biol*. 2020;65(4):45006. doi:10.1088/1361-6560/ab6e51
70. Qin Q, Shi A, Zhang R, et al. Cone-beam CT radiomics features might improve the prediction of lung toxicity after SBRT in stage I NSCLC patients. *Thorac Cancer*. 2020;11(4):964-972. doi:https://doi.org/10.1111/1759-7714.13349
71. Li H, Zhang R, Wang S, et al. CT-Based Radiomic Signature as a Prognostic Factor in Stage IV ALK-Positive Non-small-cell Lung Cancer Treated With TKI Crizotinib: A Proof-of-Concept Study. *Front Oncol*. 2020;10:57. https://www.frontiersin.org/article/10.3389/fonc.2020.00057.
72. Khorrami M, Bera K, Leo P, et al. Stable and discriminating radiomic predictor of recurrence in early stage non-small cell lung cancer: Multi-site study. *Lung Cancer*. 2020;142:90-97. doi:https://doi.org/10.1016/j.lungcan.2020.02.018
73. Farchione A, Larici AR, Masciocchi C, et al. Exploring technical issues in personalized medicine: NSCLC survival prediction by quantitative image analysis—usefulness of density correction of volumetric CT data. *Radiol Med*. 2020;125(7):625-635. doi:10.1007/s11547-020-01157-3
74. Kadoya N, Tanaka S, Kajikawa T, et al. Homology-based radiomic features for prediction of the prognosis of lung cancer based on CT-based radiomics. *Med Phys*. 2020;47(5):2197-2205. doi:https://doi.org/10.1002/mp.14104

75. Dercle L, Fronheiser M, Lu L, et al. Identification of Non–Small Cell Lung Cancer Sensitive to Systemic Cancer Therapies Using Radiomics. *Clin Cancer Res*. 2020;26(9):2151 LP - 2162. doi:10.1158/1078-0432.CCR-19-2942
76. Mazzaschi G, Milanese G, Pagano P, et al. Integrated CT imaging and tissue immune features disclose a radio-immune signature with high prognostic impact on surgically resected NSCLC. *Lung Cancer*. 2020;144:30-39. doi:https://doi.org/10.1016/j.lungcan.2020.04.006
77. Vuong D, Bogowicz M, Denzler S, et al. Comparison of robust to standardized CT radiomics models to predict overall survival for non-small cell lung cancer patients. *Med Phys*. 2020;47(9):4045-4053. doi:https://doi.org/10.1002/mp.14224
78. Botta F, Raimondi S, Rinaldi L, et al. Association of a CT-Based Clinical and Radiomics Score of Non-Small Cell Lung Cancer (NSCLC) with Lymph Node Status and Overall Survival. *Cancers*. 2020;12(6). doi:10.3390/cancers12061432
79. Kim KH, Kim J, Park H, et al. Parallel comparison and combining effect of radiomic and emerging genomic data for prognostic stratification of non-small cell lung carcinoma patients. *Thorac Cancer*. 2020;11(9):2542-2551. doi:https://doi.org/10.1111/1759-7714.13568
